# Preventable mortality in the Russian Federation: a retrospective, regional level study

**DOI:** 10.1016/j.lanepe.2023.100631

**Published:** 2023-04-19

**Authors:** Zlatko Nikoloski, Vladimir M. Shkolnikov, Elias Mossialos

**Affiliations:** a Department of Health Policy, London School of Economics and Political Science, Houghton Street, London WC2A 2AE, United Kingdom; b Laboratory of Demographic Data, Max Planck Institute for Demographic Research, Rostock 18057, Germany

**Keywords:** Preventable mortality, Russia, Sub-national analysis, Panel data analysis

## Abstract

**Background:**

Avoidable mortality, including both treatable and preventable deaths, is frequently used as an indicator of health system performance. Whilst the term treatable mortality refers to deaths that might be averted by medical interventions, preventable mortality generally reflects the impact of system-wide health policies. The concept of preventable mortality has not been evaluated extensively in the Russian Federation, particularly at the regional or sub-national (oblast) level.

**Methods:**

We calculated total preventable mortality as well as individual rates for males and females in each oblast using data from the Russian Fertility and Mortality Database (RusFMD) and computed the contributions of specific preventable causes of death to the overall rates. We also evaluated the relationship between preventable mortality and its main correlates during the years 2014–2018 using panel fixed effects modelling with variables that reflected both, behavioural risk factors and access to health care.

**Findings:**

Overall preventable mortality in the Russian Federation has been on a downward trend. Whilst 548 preventable deaths per 100,000 person-years were reported in the year 2000, only 301 per 100,000 person-years were reported in 2018. Whilst mortality due to cancer, cardiovascular, and alcohol-related diseases has declined (albeit unevenly) amongst both males and females, deaths resulting from complications of diabetes and human immunodeficiency virus infection have increased. Our findings also revealed significant heterogeneity in preventable mortality at the oblast level. For example, in 2018, deaths due to preventable causes were concentrated primarily in Siberia and the Far East. Smoking and the availability of nurses were identified as significant correlates of preventable mortality at the oblast level.

**Interpretations:**

Efforts designed to strengthen the current health care system, notably those serving the rural and less densely populated oblasts, might reduce the rate of preventable mortality in Russia. These efforts might be coupled with an ongoing focus on programs designed to reduce smoking.

**Funding:**

None.

**Research in context**

**Evidence before this study**

We employed the search terms “avoidable”, “preventable”, “amenable”, “mortality”, “Russia”, and “oblast” to search for relevant Russian and English language publications listed in PubMed and Google Scholar. We identified two publications that focused on avoidable mortality (which includes preventable and treatable mortality) evaluated on a sub-national basis in the Russian Federation. Whilst these studies provided some insight, they were descriptive in nature, focused only on the European areas of Russia, or used older and no longer useful definitions of preventable mortality. We identified no published studies that provided an in-depth assessment of preventable mortality and its main determinants at both national and sub-national levels in the Russian Federation.

**Added value of this study**

This is a quantitative and comprehensive study of avoidable mortality based on our analysis of regional oblast-level data from various sources, including the Russian Fertility and Mortality Database (RusFMD) with information focused on behavioural risk factors (e.g., smoking) from a Russia-wide household survey conducted by Rosstat. We also used the Rosstat data to evaluate alcohol sales, health system inputs, and other socioeconomic variables. We used data from all oblasts to (i) calculate male, female, and total preventable mortality and analyse existing trends between 2000 and 2018; (ii) determine the contributions of specific preventable causes to overall preventable mortality; and (iii) examine the link between preventable mortality determined at the oblast-level and its main correlates, whilst distinguishing between behavioural risk factors (e.g., smoking, alcohol use) and health system factors (health care expenditures and infrastructure).

**Implications of all the available evidence**

Overall preventable mortality has been on a downward trend in the Russian Federation. Our initial findings revealed 548 preventable deaths per 100,000 person-year in 2000 to 301 per 100,000 person-years in 2018. Whilst mortality due to cancer, cardiovascular, and alcohol-related diseases has declined (albeit unevenly) amongst both males and females, deaths due to diabetes and human immunodeficiency virus infection have increased. Our findings also revealed significant regional heterogeneity in preventable mortality; in 2018, preventable mortality was concentrated primarily in Siberia and the Far East. Similar results emerged from an evaluation of treatable mortality. Smoking and the availability of nurses were identified as significant correlates of preventable mortality at the oblast level. These findings might be addressed by focusing on improved access to preventative healthcare, particularly in rural oblasts, as well as on policies aimed at reducing smoking.

## Introduction

### Background

The political and economic transformation of the Russian Federation was unfortunately coupled with the deterioration of many social indicators.^1^ Of particular concern was the sharp and significant increase in mortality rates observed throughout the 1990s. Results of previous research revealed that most of this excess mortality was concentrated in men of working age with comparatively lower levels of education.^2^ Interestingly, excess mortality was also reported for older females, particularly women over 60 years of age.^2^ Whilst these findings could be explained in part by factors such as smoking and elevated blood pressure, alcohol consumption was identified as the chief cause of the sharp increase in mortality rates, particularly between the end of the 1980s and mid-1990s.^2^ For example, existing research^3^^,^^4^ reported a strong negative correlation between increases in life expectancy and rates of alcohol poisoning during the years 1984–2003. In addition to alcohol abuse, the inadequacies of the Russian health care system, which came to prominence in the early 1990s, also contributed to the exceptionally high rates of mortality due to otherwise avoidable causes.^5^

The chaos of the 1990s was followed by a period of rapid economic growth that commenced in the early 2000s.^6^ This period was also marked by significant reductions in mortality rates across the entire Russian Federation. These developments spurred some optimism amongst researchers and commentators who referred to this period as the beginning of a “cardiovascular revolution” in Russia.^7^^,^^8^ Changes in overall mortality rates during this period were mainly driven by reductions in deaths amongst those in the working-age population (i.e., 15–60 years of age). This change was attributed to reductions in deaths due to external causes as well as reductions in mortality associated with cerebrovascular disease amongst women.^9^^,^^10^

However, it is critical to recognise that the reductions in mortality and associated causes observed at this time were distributed unevenly across the Russian Federation. Significant reductions in mortality rates were observed primarily amongst those residing in the larger cities compared to rural oblasts and were more likely to be due to reductions in external causes rather than related to a specific impact on cerebrovascular diseases.^11^^,^^12^

Nonetheless, the precipitous reduction in mortality rates in the Russian Federation at that time was attributed to three principal factors: (i) reduced consumption of alcohol, (ii) changes in smoking habits, and (iii) the positive contributions of the Russian health care system.

### Factors associated with reductions in mortality rates

Alcohol consumption is one of the main correlates of premature mortality in the Russian Federation.^13^ Health improvements observed since the mid-2000s have been attributed in part to the strengthening of the federal alcohol policy during this period. In 2005, controls on alcohol production and sale were introduced that dramatically accelerated the removal of smaller commercial alcohol producers from the Russian market. Steps were also taken to ensure full denaturation of alcohol contained in various non-beverage products, for example, hygienic solutions, cleaning agents, and eau de cologne formulations. Whilst there were no systematic efforts in place to document the effectiveness of these policies, their introduction was coupled with a significant reduction in alcohol poisoning.^4^^,^^14^ Some of these policy-based restrictions were also accompanied by a shift in the types of alcohol products consumed and the new-found popularity of drinks with a lower alcohol content (e.g., beer).^4^ Furthermore, previous research studies also highlighted an apparent increase in the number of people who abstained from alcohol consumption as well as reductions in the overall quantity of alcohol consumed during this period (2006–2017), particularly amongst the younger members of the population.^15^

The remarkable reduction in mortality rates observed during this period was also attributed, at least in part, to a reduction in the prevalence of smoking. Estimates from the late 1990s suggested that 40–70% of males and 5–20% of females were active smokers at that time.^16^^,^^17^ Since that time, there has been an overall reduction in smoking, albeit more significantly amongst men and women at ages under 45. This is particularly the case since 2008 when Russia ratified the Framework Convention on Tobacco Control.^18^ Additional legislation passed in 2013 imposed a comprehensive ban on smoking in indoor workplaces, indoor public places, and on public transport, and prohibited advertising, promotion, and sponsorship of tobacco.^18^ Health warnings on tobacco products were also introduced at this time, together with an increase in the tobacco tax, currently at 51%.^18^ Largely as a result of these policies, the prevalence of smoking has decreased, albeit more substantially and consistently amongst males than females. Data from the Russia Longitudinal Monitoring Survey - Higher School of Economics (RLMS-HSE) revealed that the age-adjusted prevalence of smoking amongst males decreased from 61 to 63% in 1996–2007 to 48% in 2016. Whilst smoking amongst females initially increased from 12% in 1996 to 19% in 2007, this value decreased slightly to 17.5% in 2016.^19^

The aforementioned alcohol and tobacco control policies were accompanied by improvements in the health care system, which led to further reductions in mortality rates. In 2006, Russia introduced an ambitious and multi-purpose project entitled “Health” which provided the national health care system with direct subsidies from the national budget.^20^^,^^21^ The project had four main priorities: (i) improvement of primary care, including (beginning in 2006) higher salaries for general practitioners employed in polyclinics; (ii) strengthening of the emergency care infrastructure; (iii) promotion of high-technology medical care; and (iv) construction of new federally-funded medical centres. Although the impact of this project on health and health care throughout Russia has not yet been evaluated objectively, this increased investment may have had a positive impact on health outcomes.^7^^,^^9^ Since the mid-2000s, a larger proportion of both men and women have initiated treatment with anti-hypertensive drugs. This has been associated with a net decrease in blood pressure amongst women, which reduces their risk of death due to cardiovascular diseases.^22^ In addition, medical procedures that are relevant for preventable mortality have been spreading across the Russian regions. Namely, these are coronary bypass and stenting surgery as well as PCIs (percutaneous coronary interventions), ultimately resulting in improved health outcomes.^23^^,^^24^

### Avoidable and preventable mortality: evidence from the literature

Interactions amongst the policies outlined above should result in a substantial reduction in overall mortality rates, including mortality due to causes identified as preventable. The term “preventable mortality” is the most common metric used to gauge the impact of system-wide health policies. This term is included as part of the wider concept of avoidable mortality, which includes both amenable (also known as treatable) and preventable causes of mortality.^25^, ^26^, ^27^, ^28^ However, whilst treatable mortality focuses on the analysis of causes of death that might be averted by appropriate medical intervention, preventable mortality is a broader concept that also includes deaths that could have been avoided by the implementation of critical public health interventions, specifically those focused on wider determinants of health, including behavioural, lifestyle, environmental, and socioeconomic factors.^29^^,^^30^

Factors contributing to preventable mortality in the Russian Federation have not been fully evaluated, particularly at the sub-national, or oblast level. Sabgayda et al.^31^ provided a descriptive analysis and some simple correlations that linked this concept to regional levels of economic development and poverty rates in the Russian Federation. Likewise, Andreev et al.,^5^ focused exclusively on treatable mortality during the first few years of the transition process (i.e., the 1990s) and concluded that deaths resulting from treatable causes had an increasing role in reducing life expectancy in Russia. Most recently, Ivanova et al.^32^ evaluated the contributions of health care to the overall reduction in mortality rates. Whilst these studies have provided significant insight into the impact of both preventable and treatable causes of mortality, they are either descriptive in nature, use older definitions of preventable mortality,^33^ or focus only on the European regions of Russia. Moreover, the study by Ivanova et al.^32^ includes calculations based on an upper age limit of 65 years. Currently, 75 years is the internationally-set upper limit for these types of calculations; the 10-year age difference limits the use of these data for cross-country comparisons. In addition, the study published by Ivanova et al.^32^ did not consider critical determinants of avoidable mortality, for example, the contributions of behavioural factors (e.g., smoking and alcohol use).

There are currently no in-depth studies or sources of information that focus on preventable mortality at the national and sub-national levels in the Russian Federation, nor any quantitative assessments of their main determinants. More specifically, our study has the following aims/objectives:

(i) To estimate rates of preventable mortality in the Russian Federation and trends for males and females at national and sub-national (oblast) levels for the years 2000–2018;
(ii) To estimate the contributions of various causes of death to overall rates of preventable mortality for both males and females;
(iii) To estimate the rates of treatable mortality (including the contributions of different causes of death to overall treatable mortality); and
(iv) To examine the link between preventable mortality and its determinants for the years 2014–2018 based on data pertaining to the entire Russian Federation. We focus specifically on the contributions of behavioural risk factors and health care system-related factors.

## Methods

### Datasets

Three data sources were used in this analysis. The Russian Fertility and Mortality Database (RusFMD) of the Center for Demographic Research at New Economic School^34^ was the source of information used to estimate preventable and treatable mortality in the Russian Federation at the oblast level. This database includes mortality indicators from the Russian Federation and its regions starting in 1959 that were calculated from official statistical data. The data obtained from the RusFMD are unique, have not been published elsewhere, and are based fully on data from the Federal State Statistics Service of the Russian Federation.^34^ Although the dataset includes its own coding system, the information is closely aligned with standard ICD-10 codes. Furthermore, RusFMD contains mid-year population estimates used to calculate fertility and mortality rates. Population exposure estimates in 1- and 5-year groups are available across regions of Russia and in Russia as a whole beginning in 1989.^34^ Findings are listed by gender which facilitated our calculation of gender-specific mortality rates.

Data focused on risk factors, including smoking, were obtained for the years 2014, 2016, and 2018. These data came from a survey entitled “Итоги комплексного наблюдения условий жизни населения” (translated: “Results of a comprehensive observation on living conditions of the population”) conducted by Rosstat that included 60,000 households in all oblasts. The goal of this survey was to obtain statistical information on living conditions in the Russian Federation. The quality of official survey/census data in the Russian Federation has been commended by international sources.^35^ The survey was conducted three times (years 2014, 2016, and 2018 as noted above) on a representative sample of the entire population of the Russian Federation as well as at the oblast level. The survey also includes information related to health care including the prevalence of smoking and alcohol use and the frequency at which health care facilities were used.^36^ From this survey, we distilled a single variable that defined the prevalence of smoking via a combination of past, occasional, and daily tobacco use that captures the overall burden of smoking. Of note, the prevalence of smoking reported in this survey is somewhat lower than that derived from RLMS data.^22^ However, this variable captures both, present and past smoking habits, and it is thus a useful addition to our modelling exercise. These findings have already been summarised at the oblast level on the Rosstat website.^36^ However, because these summaries only provide information on the frequency of alcohol consumption for two of the three years, we used a different proxy to capture the prevalence of alcohol use, as explained further below.

Finally, data series from the regional Rosstat repository were used as additional variables to model correlates of preventable mortality in the Russian Federation.^37^ The variables include the level of economic development and availability of health care infrastructure, amongst others. Further details on the specific series are presented in the Supplementary Materials. The regional statistics also include data on total sales of vodka per oblast; this value was expressed as a share of total alcohol sales.^37^ As indicated by existing research, excess mortality in Russia is related to the binge drinking of hard liquors, rather than wine or beer.^13^^,^^38^ Previously, these statistics were used by international researchers^31^^,^^32^^,^^39^ as well as by numerous international organisations.^40^^,^^41^ The data featured in this manuscript were obtained before February 2022 and are not currently accessible due to the ongoing conflict in Ukraine.

### Statistical analyses

#### Estimating preventable and treatable mortality

We used several resources to estimate age-standardised preventable and treatable mortality including (i) data on deaths by age and associated underlying causes of death from the RusFMD; (ii) data on population exposure by age for computation of death rates; and (iii) the average age of the European population in 2013.^42^

To classify deaths as preventable or treatable, we relied on the list provided by Eurostat/OECD.^43^ Preventable mortality is defined as all causes of death that can be prevented through effective public health and primary interventions (i.e., those that can be introduced before the onset of disease). By contrast, treatable mortality is defined as all causes of death that are amenable to medical intervention (i.e., deaths that can be averted by appropriate medical/surgical care). To facilitate the identification of preventable or treatable deaths, the upper age limit was set to 75 years^43^ which is also consistent with the existing literature on this subject in Russia.^5^ We conducted a sensitivity analysis in which we reduced the upper age limit to 70 and 65 years and, as anticipated, we found that this resulted in substantially different outcomes compared to findings obtained using the canonical upper age limit of 75 years. A full set of conditions and diseases included as preventable or treatable are included in Appendix Tables A1 and A2, respectively.

The causes of death were classified according to the International Statistical Classification of Diseases and Related Health Problems, 10th Revision.^44^ The RusFMD coding system is closely aligned with the ICD-10 codes (see Appendix Table A3). Age-standardised preventable mortality rates (PMRs) by oblast and year are calculated using the following equation:

(1) $${PMR}_{i}=\sum_{x}\left(\frac{D_{x,i}}{P_{x}}.\theta_{x}^{s}\right)$$

where, PMR is the preventable mortality rate; *D*_x,i_ is the number of deaths in age group x from preventable causes i; P_x_ is the population exposure in age group x; and $\theta_{x}^{s}$ is the standard population weight of the age group x. An analogous formula was used to estimate treatable mortality. Age-standardised mortality rates were calculated for the entire country as well as at the sub-national (oblast), including overall values as well as those disaggregated by gender. A full list of oblasts is provided in Appendix Table A4. As shown, the RusFMD database is closely aligned with ICD-10 codes, except in specific instances in which some of the causes of death were included within larger groups that were labelled “other”. To avoid inflating the death count inappropriately, these causes of death were excluded from the analysis. Furthermore, the fact that these categories were included in larger groups with other causes of death suggests that they are most likely few in number. Thus, this decision should not have a significant impact on the overall validity of the results. Further details regarding these adjustments are provided in the Supplementary Materials.

#### Analysis of correlates of preventable mortality

In addition to our evaluation of preventable mortality at national and oblast levels, we also estimated the link between the rates of preventable mortality with other factors, including those related to health system supply. We relied on a panel fixed effects whilst also controlling for year effects. We constructed a panel data set for all oblasts for each of the 3 years (2014, 2016, and 2018) for the 83 oblasts in the Russian Federation as defined by the Russian constitution before 2014. We used the following equation to estimate the overall preventable mortality:

(2) $$\ln\left({PMR}_{kt}\right)=\beta_{0}+\beta_{1}X_{kt}+\beta_{2}Y_{kt}+\varepsilon_{kt}$$

where the natural logarithm (ln) of the age-adjusted preventable mortality, PMRkt in oblast *k* at time *t* is determined as a function of the risk factors (X) and health system supply factors (Y).

The natural log of the total preventable mortality derived as described above was introduced as the dependent variable. The independent variables included two sets of predictors: (i) behavioural risk factors (i.e., the prevalence of smoking and alcohol use as described above) and (ii) health care system-associated variables (i.e., the number of physicians, nurses, and hospital beds per 10,000 inhabitants and the health expenditure as a share of Gross Regional Product [GRP]). We also controlled for the following variables: (i) level of economic development (proxied by GRP per capita, adjusted for inflation), (ii) regional population density, (iii) the proportion of the population residing in an urban setting, (iv) the overall poverty rate, and (v) the ratio of females to males. A table that includes the definitions of the variables used in the model is included in the Supplementary materials – Appendix Table A5.

We used panel fixed effects to estimate the first model (Eq. 2). We assumed correlations between several of the independent variables as well as the time-invariant portion of the error term; thus, ordinary least squares estimates would be inconsistent. By contrast, panel fixed effects would permit us to remove most endogenous effects whilst providing consistent estimates for other, only mildly endogenous variables that varied with time. Whilst most panel data studies take into account the possibility of reverse causality, we assumed that preventable mortality alone would have no significant reverse impact on the explanatory factors selected. Likewise, as our panel covers a very brief period (i.e., 2014–2018), it is unlikely that preventable mortality could contribute to significant changes in both behavioural risk factors and health care system variables. This is particularly the case for the health care system variables, given the time that would be needed to increase the availability of both health care facilities and personnel.

As a robustness check, we repeated this analysis whilst using treatable mortality as a dependent variable with health care system supply-side factors (healthcare infrastructure and expenditures) as the only independent variable (the results are featured in the Supplementary Materials). All analyses were performed in Stata 14.^45^

### Role of the funding source

None.

## Results

**Fig. 1:**
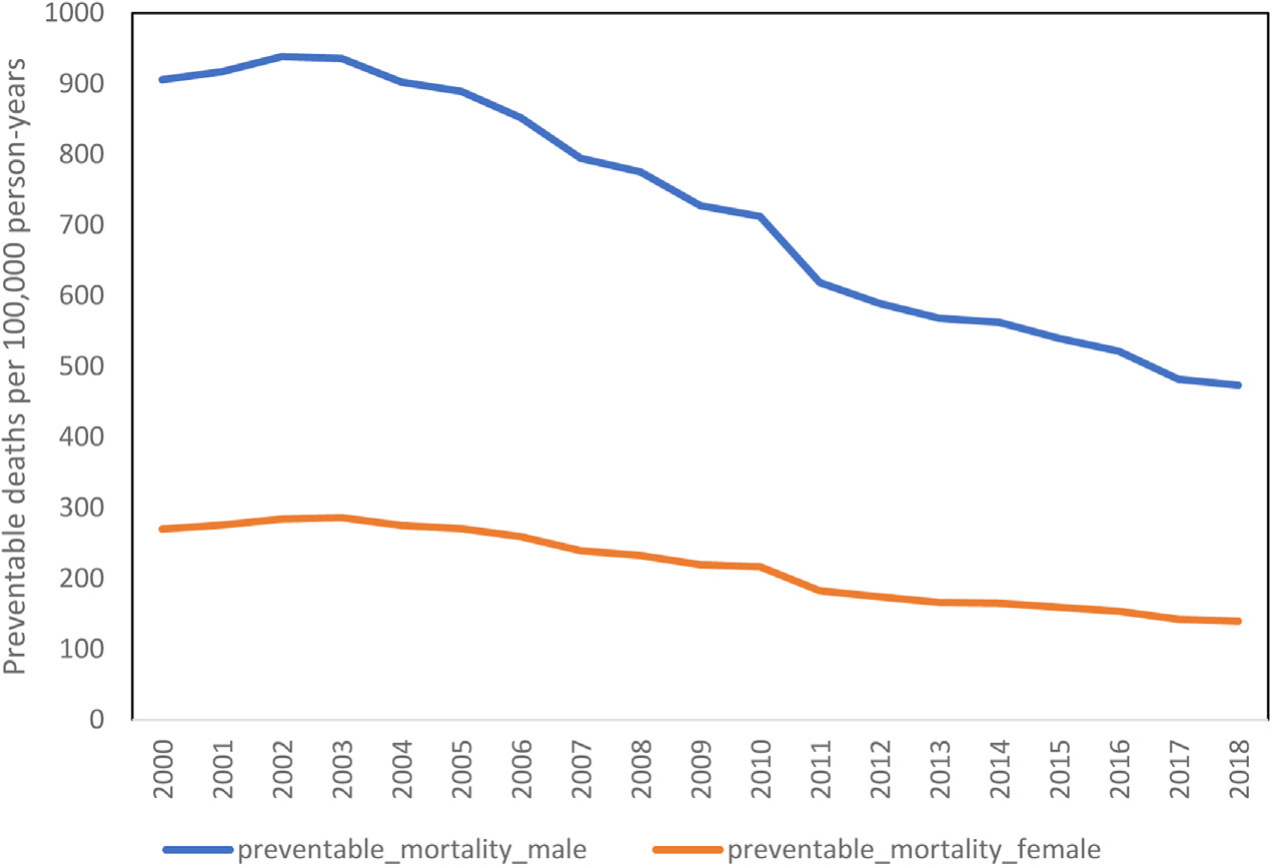
**Preventable mortality rates (PMRs) in the Russian Federation.** Data are presented as preventable deaths for males (blue line) and females (orange line) per 100,000 person-years.

*Sources:* Russian Fertility and Mortality database (RusFMD), OECD/Eurostat, and the authors' calculations.

Fig. 1 summarises the trends in overall preventable mortality for males and females in the Russian Federation. As shown, both upward trends ceased in 2002; since that time, preventable mortality for both males and females has assumed a downward trend. Preventable mortality rates (PMRs) for males dropped from 905.8 deaths per 100,000 person-years in 2000 to 473.3 deaths per 100,000 person-years in 2018, representing a reduction of 47.8%. Similarly, PMRs for females dropped from 269.7 deaths per 100,000 person-years in 2000 to 139.5 deaths per 100,000 person-years in 2018. This downward trend is also evident in the overall rates of treatable mortality (Appendix Fig. A1). As discussed in the Methods section, we performed an additional robustness check in which we reduced the upper age limit for these mortality calculations by five and ten years. This resulted in a significant underestimation of both preventable and treatable mortality (Appendix Table A6). An additional robustness check was performed that compared the trend of the preventable and treatable mortality rates with those due to causes that are not considered preventable and treatable, respectively (Appendix Figs. A2 and A3). The main conclusion from this analysis is that preventable and treatable mortality rates have been dropping at a marginally higher rate compared to mortality associated with other causes of death.

Table 1 summarises preventable mortality associated with specific causes of death as well as the contributions made by these causes to the overall gender-specific PMRs in both 2000 and 2018. Our findings reveal that ischemic heart disease (IHD) was the cause of 182.6 deaths per 100,000 person-years and thus the most prominent source of preventable mortality reported for males in 2000. This was followed by cerebrovascular disease (99.5 deaths per 100,000 person-years), and alcohol-related causes (80.9 deaths per 100,000 person-years). Changes in cause-specific preventable mortality amongst males varied between 2000 and 2018. IHD as a cause of preventable mortality amongst males dropped by 43% from the aforementioned 182.6 deaths per 100,000 person-years in 2000 to 103.3 deaths per 100,000 person-years in 2018. Similar reductions were observed with respect to alcohol-related deaths amongst males. Male deaths attributed to alcohol-related causes dropped by 33 percent from 80.9 per 100,000 person-years in 2000 to 53.5 per 100,000 person-years in 2018. Of note, the 2016 version of the Russian nomenclature included several new alcohol-related causes of death; to maintain meaningful comparisons over time, we included only those causes of death that appeared in the initial 2000 version. Despite these advances, not all preventable deaths were reduced during this study period. For example, preventable mortality amongst males due to human immunodeficiency virus (HIV)-related causes increased dramatically from 0.2 per 100,000 person-years in 2000 to 18.5 per 100,000 person-years in 2018. Our findings also reveal increases in diabetes-related deaths as well as increases in their overall contribution to preventable mortality amongst males. Whilst similar findings emerged with respect to female PMRs, these rates have remained markedly lower, due at least in part to reductions in preventable mortality associated with conditions such as cardiovascular disease and lung cancer. We obtained similar findings in our analysis of contributing causes to treatable mortality (Appendix Table A7).

**Table 1:** Preventable mortality and contributing causes per 100,000 person-years (male and female).

Males					
Preventable mortality (per 100,000 person-years)			Contribution to preventable mortality (%)		
	2000	2018		2000	2018
Preventable infectious diseases	6.1	1.7	Preventable infectious diseases	0.7	0.3
HIV/AIDS	0.2	18.5	HIV/AIDS	0.0	3.7
Hepatitis	0.8	1.8	Hepatitis	0.1	0.4
Tuberculosis	19.5	4.6	Tuberculosis	2.2	1.0
Preventable cancers (other than lung cancer)	72.2	48.2	Preventable cancers (other than lung cancer)	8.0	9.7
Lung cancer	76.5	48.9	Lung cancers	8.4	9.8
Vehicular accidents	41.5	19.5	Vehicular accidents	4.6	3.9
Assault	44.4	8.3	Assault	4.9	1.7
Alcohol-related deaths	80.9	53.5	Alcohol-related deaths	8.9	11.3
Diabetes	2.5	6.3	Diabetes	0.3	1.3
Ischemic heart disease	182.6	103.3	Ischemic heart disease	20.1	20.8
Hypertensive disorders	7.8	3.1	Hypertensive disorders	0.9	0.6
Cerebrovascular diseases	99.5	45.3	Cerebrovascular diseases	11.0	9.1

Females					
Preventable mortality (per 100,000 person-years)			Contribution to preventable mortality (%)		
	2000	2018		2000	2018
Preventable infectious diseases	3.6	1.1	Preventable infectious diseases	1.3	0.7
HIV/AIDS	0.0	8.7	HIV/AIDS	0.0	5.8
Hepatitis	0.4	0.9	Hepatitis	0.2	0.6
Tuberculosis	2.3	0.9	Tuberculosis	0.8	0.6
Preventable cancers (other than lung cancer)	21.5	13.5	Preventable cancers (other than lung cancer)	8.0	8.8
Lung cancer	6.8	6.6	Lung cancers	2.5	4.3
Vehicular accidents	12.1	5.7	Vehicular accidents	4.5	3.7
Assault	12.5	2.3	Assault	4.6	1.5
Alcohol-related deaths	26.7	21.4	Alcohol-related deaths	9.9	15.3
Diabetes	3.6	6.1	Diabetes	1.3	4.0
Ischaemic heart disease	60.9	31.4	Ischaemic heart disease	22.6	20.6
Hypertensive disorders	5.2	1.6	Hypertensive disorders	1.9	1.0
Cerebrovascular diseases	57.5	19.7	Cerebrovascular diseases	21.3	13.0

**Fig. 2:**
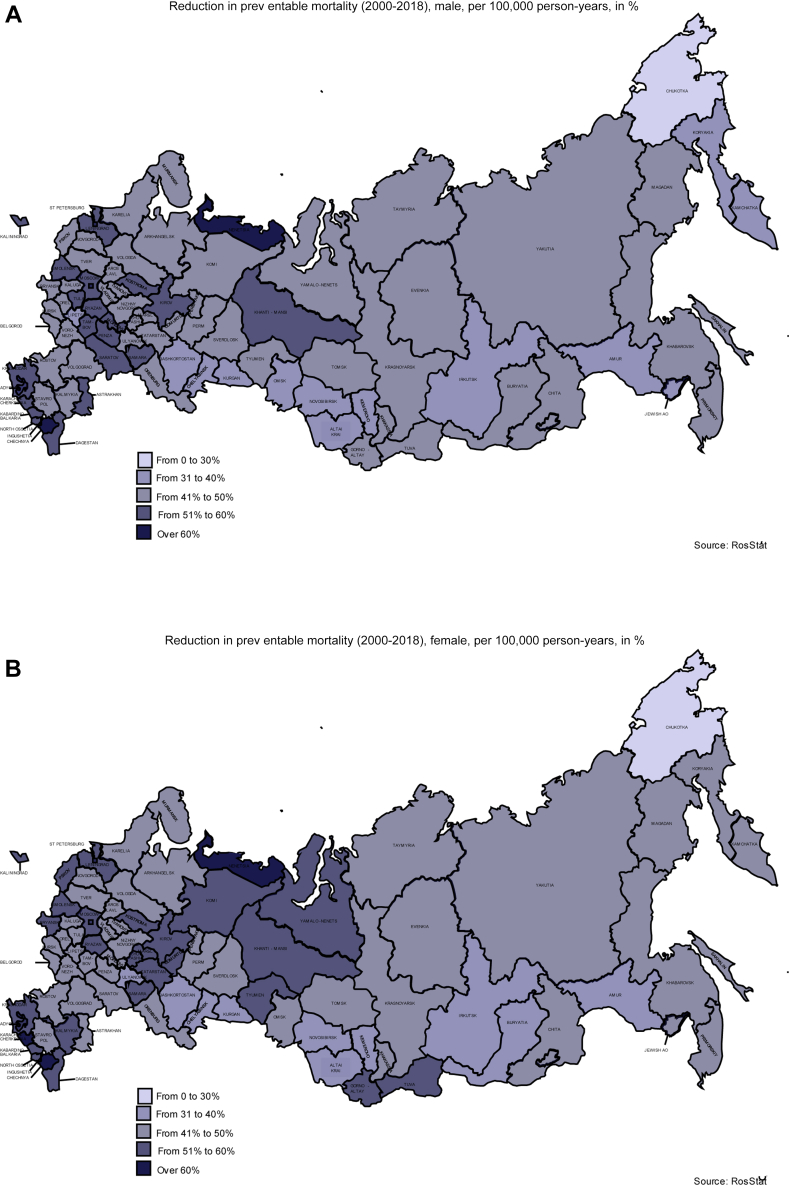
**Reduction in preventable mortality at the subnational level.** Shown are data for males (A) and females (B) in the Russian Federation. Data are presented as percentage change.

*Sources:* Russian Fertility and Mortality database (RusFMD), OECD/Eurostat, and the authors' calculations.

Findings shown in Fig. 2A document the change in PMRs between 2000 and 2018 across all oblasts in Russia for males. Whilst the results reveal a reduction in male PMRs over the study period, the rate of change varied amongst the oblasts. Whilst PMRs amongst males dropped by more than 50% in Ryazan and Saint Petersburg, reductions of less than 33% were reported in Amurskaya Oblast and Altayskii Kray. Overall, preventable deaths remain more heavily concentrated in the non-European parts of the Russian Federation. In 2018, the five oblasts with the highest rates of male preventable mortality were in Siberia (Irkustkaya Oblast, Respublika Tyva, and Zabaykalskiyi Kray) or the Far East (Chukostkii Avtononmii Okrug and Everyskaya Avtonomnaya Oblast) (Appendix Fig. A4A). Similar findings emerge from our evaluation of female preventable mortality (Fig. 2B and Appendix Fig. A4B). As a robustness check, we repeated the same exercise using overall preventable mortality; this evaluation yielded results that were consistent with the gender-specific PMRs presented above (see Appendix Figs. A5 and A6). As a second robustness check, we repeated the same exercise with a focus on treatable mortality. The results of this second check also revealed an East/West divide that was similar to that observed for preventable mortality (Appendix Figs. A7–A11).

Furthermore, to expand on the results of the comparison of preventable or treatable causes of mortality with that due to other (residual) causes, we conducted a sub-national analysis in an attempt to correlate the extent of preventable and treatable mortality with mortality due to causes not considered preventable and treatable, respectively (Appendix Figs. A12 and A13). Overall, our findings highlighted a strong correlation between these series, given the downward trend of mortality in the Russian Federation.

Table 2 presents the findings from the panel fixed effects analysis using the natural logarithm of total overall preventable mortality as a dependent variable. We present the results using two principal sets of independent variables: (i) behavioural risk factors and (ii) health care system inputs introduced separately (Model 1 and Model 2) and then together (Model 3). The three models also include an extensive set of control variables. The full results of this analysis are reported in Appendix Table A9. The main conclusions that can be drawn from this analysis are broadly consistent across all three models. First, our findings reveal that smoking is a statistically significant correlate of overall preventable mortality, although only when including the risk factors and control variables. As shown, a single percentage point increase in the fraction of adults who smoke is associated with a 0.3% (95% confidence interval [CI], −0.04 to 0.6) increase in overall preventable mortality. Second, the availability of nurses (number per 10,000 inhabitants) is significantly inversely associated with overall preventable mortality; a single unit increase in the number of nurses per 10,000 inhabitants is associated with a 0.3% (95% CI, −0.6 to 0.05) decrease in total preventable mortality. To maintain consistency, we have applied the same model to account for the health care system supply variables with treatable mortality as a dependent variable. As shown in Appendix Table A10, the results of this analysis yielded no statistically significant links between treatable mortality and variables associated with health care system supply.

**Table 2:** Fixed effects regression analysis of contributing causes of preventable deaths (2014–2018) with the log of preventable mortality used as the dependent variable.

Variable	Model 1	Model 2	Model 3
Sales of vodka (in % of total alcohol sales)	−0.008 [−0.003 to 0.001]		−0.009 [−0.002 to 0.001]
Smoking, prevalence, % of the adult population	0.003∗ [−0.0003 to 0.006]		0.003 [−0.0007 to 0.006]
Physicians per 10,000 inhabitants		−0.00003 [−0.004 to 0.004]	−0.00002 [−0.004 to 0.004]
Hospital beds per 10,000 inhabitants		0.001 [−0.007 to 0.004]	0.001 [−0.008 to 0.004]
Nurses per 10,000 inhabitants		−0.003∗ [−0.06 to 0.001]	−0.003∗ [−0.006 to 0.0002]
Health expenditures, % of the gross regional product (GRP)		0.001 [−0.009 to 0.012]	0.001 [−0.009 to 0.011]
Intercept	5.02∗∗∗ [2.41–7.63]	4.35∗∗∗ [2.24–6.47]	4.04∗∗∗ [1.83–6.25]
R-squared	0.078	0.104	0.070
Number of observations	248	249	248
Year effects	Yes	Yes	Yes
Number of groups	83	83	83

∗∗∗*p* < 0.01, ∗*p* < 0.10. The values presented are parameter estimates from respective regression analyses. All models are estimated and include robust standard errors which are shown in the brackets.

The models used in this analysis also controlled for the following variables: Gross regional product (GRP) per capita, the density of population per km^2^, the fraction of the population living in urban settings, poverty rates, and the ratio of females to males in the population. Model 1 includes only risk factors; Model 2 includes only health system variables; Model 3 includes all variables. The modelling exercise was based on a panel of Russian oblasts for the years 2014, 2016, and 2018.

## Discussion

In this study, we evaluated rates of preventable and treatable morality and their determinants with respect to Russia as a whole, as well as in individual regions of the Russian Federation. We found that preventable and treatable mortality rates for both males and females have diminished between 2000 and 2018. Whilst our data revealed similar reductions in the rates of preventable deaths due to cardiovascular disease and alcohol, mortality due to diabetes and HIV infection has increased amongst both males and females. We also noted significant heterogeneity in the rates of both preventable and treatable mortality across Russian oblasts, with the highest number of preventable deaths per capita in 2018 concentrated primarily in Siberia and the Far East. Finally, the results of our analysis of sub-national panel data suggest that both smoking (a behavioural risk factor) and the availability of nurses (a proxy for public health care development) are significant determinants of preventable mortality in the Russian Federation.

Whilst preventable mortality has been on a downward trend since 2002, Russia still lags behind many of the high-income Organisation for Economic Cooperation and Development (OECD) countries. The overall PMR of 301.3 per 100,000 person-years reported for Russia in 2018 was substantially higher than the OECD average of 142 preventable deaths per 100,000 person-years. This rate was also higher than those reported for many of the lower-performing OECD countries including Latvia (256 per 100,000), Hungary (243 per 100,000), and Lithuania (226 per 100,000).^46^ Amongst all OECD nations, Israel reported the lowest PMR in 2018, at 73 per 100,000.^46^ Interestingly, the 2018 PMRs in Russia are comparable to those reported for the most economically deprived areas in England.^47^

Despite a slight increase, mortality associated with diabetes in Russia remains lower compared to rates reported for other upper-middle-income countries, for example, Mexico.^48^ The observed increase in diabetes-related mortality may be due in part to economic growth experienced by Russia over the last several decades.^6^ It may have also been driven by changes in the registration and coding practices used to diagnose and report diabetes in the Russian Federation. By contrast, whilst the HIV epidemic had been concentrated in a few marginalised groups in previous years, it has recently spread to the general population, with heterosexual transmission emerging as the predominant source of new infections.^49^^,^^50^

Our study revealed two critical correlates of preventable mortality. Amongst our statistically significant findings, results from the panel data analysis revealed that a single percentage point increase in the prevalence of smoking was associated with an 0.3% increase in the overall PMR (NB: As described in the Methods, our smoking variable includes both a present and a past history of smoking). This finding is consistent with previous reports and existing knowledge regarding the impact of smoking on overall mortality in the Russian Federation.^19^^,^^51^ Data collected internationally have focused primarily on the impact of smoking on deaths as they relate to cardiovascular disease. For example, Ezzati et al.^52^ using global data, reported that one in ten deaths due to cardiovascular disease could be attributed to smoking. Other studies have documented that smokers are at higher risk for premature mortality, particularly from cancer, heart disease, and respiratory disorders.^53^^,^^54^ Similarly, the existing literature has documented that reducing smoking or quitting smoking altogether results in a rapid reduction in the risk of developing CVD.^55^ We also found that the state of health care infrastructure (specifically, the number of nurses per 10,000 inhabitants) was significantly inversely associated with preventable mortality. Our data revealed that an increase in the availability of nurses was associated with lower rates of preventable mortality. The availability of nurses may lead to the increased use of preventative health care services and thus improved health outcomes.^56^ Previously and in the context of the United States, it has been shown that the density of nurses is associated with the reduction in preventable and overall mortality.^57^, ^58^, ^59^ Similar findings have emerged from other high-income countries.^60^^,^^61^ Nurses are usually involved in early detection and prompt intervention, which is particularly important in short term (and corresponds with the setting of our study). In addition, this finding could be a direct reflection of the lower density of nurses in some of the oblasts in the Siberian and Far East Federal Districts. As the overall preventable mortality is due to causes that could be addressed by strong primary healthcare,^62^^,^^63^ we do not find statistical significance for the density of physicians and hospital beds. Whilst the density of doctors may be associated with a longer-term reduction in mortality, this falls outside of the scope of this paper. Furthermore, this variable could be somewhat biased as it includes doctors of all specialties (e.g., hospital-based, ambulatory care, and others). Historically, Russia has experienced a deficit of physicians. Despite efforts to increase their overall number, a weak primary healthcare system has led to an increase in the number of doctors working in hospital settings.^64^ At the same time, the healthcare system remains heavily hospital-focused with an overall number of bed-days per capita that is 70–75% higher than in the EU.^65^ Furthermore, we do not find evidence that our proxy variable for alcohol consumption was associated with a reduction in overall preventable mortality. This finding could be attributed to three things. First, the modelling exercise encompasses a comparatively short period (2014–2018), which precludes adequate inclusion of alcohol-related conditions that take many years to develop (e.g., cirrhosis). Second, the stock of alcohol-related mortality has already been significantly reduced, putting the centre of gravity on high mortality from CVD at ages 50–80.^10^ Third, this finding could also be due to the reliability (or lack thereof) of some of the nationally-published data on alcohol consumption in Russia. For example, a recent WHO report reported a much more conservative reduction in alcohol consumption compared to the official data published by Rosstat.^66^

Our findings also revealed substantial geographic heterogeneity. Whilst there has been some reduction in overall PMRs between the years 2000 and 2018, progress has been uneven and some oblasts have fallen behind. In 2018, the five oblasts with the highest PMRs were located in two Federal Districts in non-European Russia, specifically, Siberia and the Far East. Similar findings emerged in an evaluation of treatable mortality. Amongst the problems in these oblasts, many include vast land areas with comparatively low population densities. This can result in significant transport-related challenges that limit access to health care services.^37^ Furthermore, alcohol consumption has increased in some of these oblasts (e.g., Chukotka) coupled with an increase in the prevalence of occasional smoking (e.g., Amurskii Krai, Zabaikalskii Krai). Previous studies in other countries as well as those focused on Russia revealed that measurable reductions in mortality rates are detected more frequently in larger cities compared to smaller towns and rural settlements.^11^^,^^12^

Finally, our findings provided no evidence to suggest that spending on health care was directly associated with the overall levels of preventable or treatable mortality. This may be due in part to the way in which resources for health care are allocated in Russia, including funds from the central authority to the oblasts as well as the disposition of funds within individual oblasts. In previous research, investigators have noted that resource allocation for health care was heavily centralised and that the roles and unique needs of individual communities were not addressed.^20^ In many cases, patients with severe life-threatening diseases who resided in peripheral oblasts needed to travel a great distance to medical centres in Moscow, Saint Petersburg, or Novosibirsk, amongst other major cities. It is important to note that mortality estimates depend upon the quality of the underlying data. Historically, there have been some quality issues associated with the Russian mortality data. Efforts have been made to reconstruct data series going back to 1965, although the quality of data before 1970 remains questionable.^67^ Some of the issues with data quality have been discussed extensively in previous work.^4^^,^^10^^,^^32^ However, in our analysis, we rely on data collected from the early 2000s and onwards, at a time at which the data quality has been considered high.^34^ More specifically, RusFMD data were used extensively in the Global Burden of Disease study on Russia as well as for subsequent studies that have built on this research.^68^, ^69^, ^70^

There are some limitations to our findings. First, despite the high quality of data collected in the Russian mortality database, there were several instances in which it was impossible to perform one-to-one matching with the ICD-10 codes. However, the few adjustments that needed to be made should not have a significant impact on the overall results. In some of these instances, deaths were aggregated in groups categorised as “other”, most likely due to a low overall prevalence and thus with little to no impact on the overall validity of the results. Second, whilst it could be argued that additional variables might be included in the model (i.e., average educational attainment per oblast), the final set of variables was chosen based on findings that were available in the regional Rosstat database. When using short time series, there is some risk of obtaining spurious correlations. Thus, some caution should be exercised when interpreting the findings. Furthermore, given the low value of the R^2^ in the panel data analysis, much of the cause for the decline of preventable mortality remains unknown. The fairly short time span used in the modelling exercise (2014–2018) partly explains the lower explanatory power of the model. However, it remains difficult to amass sufficient high-quality oblast-level data for a longer period in the context of the Russian Federation.

Importantly, whilst the international literature establishes a robust link between the quality of healthcare and preventable/avoidable mortality,^71^^,^^72^ the lack of data on the quality of the Russian healthcare system, prevented us from including it in the modelling exercise. As such, our model captures only the crude availability of basic healthcare resources and does not account for the quality of healthcare, which has been a perennial problem in Soviet/post-Soviet Russia. The existing evidence, where available, is scant and highlights only a few specific interventions for the treatment of ischaemia which experienced very substantial improvement over the late 2000s and the 2010s.^23^^,^^24^ Thus, some caution should be exercised when interpreting the findings of this modelling exercise. It is important to note that, whilst the quality of the Russian mortality data is generally strong, there are occasional challenges with the post-mortem diagnostics and cause of death coding. For example, evidence points to inconsistency in coding across Russian oblasts, making Russia standing out in comparison to other countries.^73^ This is coupled with some temporal changes in practices for cause-of-death coding at the all-Russia level, particularly circulatory diseases due to possible exchanges with senility, diabetes, and dementia over the late 2000s and the 2010s.^74^ However, as we focus on deaths between the ages of 0 and 75, we believe that some of these problems are mitigated as they tend to concentrate at old ages. Finally, because our analytical model establishes association, rather than direct causal links, some caution is advised when interpreting the results.

## Conclusion and policy implications

Several conclusions can be drawn from this research. First, our findings highlight the steady reduction in preventable mortality between the years 2000 and 2018 which was driven primarily by reductions in mortality due to lung cancer, cardiovascular disease, and alcohol-related disorders. However, these reductions have been uneven across oblasts and across causes that contribute to total preventable mortality. In 2018, the highest concentrations of preventable deaths were reported in several oblasts in Siberia and the Far East. In addition, further reductions in preventable mortality from these causes have been counterbalanced in part by a rise in deaths associated with diabetes and HIV infection. Finally, the results of our analysis revealed that behavioural risk factors (e.g., smoking) and environmental factors (e.g., health care infrastructure, specifically the availability of nurses) were statistically significant correlates of overall preventable mortality in 2014–2018. Nevertheless, some caution should be exercised when interpreting our findings as, due to a lack of data, our modelling exercise does not account for the quality of care. As indicated in the international literature, the quality of healthcare accounts for a significant reduction in preventable and treatable mortality worldwide.

The results of our study suggest the value of further efforts to address smoking and strengthen the health care system. In recent years, Russian authorities have been taking steps in that direction. For example, the Russian Ministry of Health has issued regulations that specify the maximum allowable distance between residential areas and medical facilities.^75^ Furthermore, central authorities have also made some efforts to improve the organisation of service delivery in the polyclinics with the goal of improved access and reductions in waiting times.^76^ In addition, given the lack of data on the quality of healthcare, using complementary research techniques (e.g., qualitative research) might be useful in the effort to identify the causes of the reduction in preventable and treatable mortality.

Unfortunately, many of these positive changes were followed by a period of extraordinarily high rates of excess mortality and reductions in life expectancy due to the COVID-19 pandemic in Russia.^77^^,^^78^ The general weakness of the Russian health care system most likely contributed to the explosion in premature mortality in 2020–2021. These factors, together with the current geopolitical crisis in Russia, will most likely limit further reductions in both overall and preventable mortality throughout the Russian Federation.

## Contributors

The study was conceived by Z.N. and E.M. Z.N. conducted the analysis with inputs from E.M. and V.M.S. E.M. and V.M.S. verified the statistical analysis. The paper was drafted jointly by all authors. All authors have seen and approved the final version of this draft.

## Data sharing statement

Data on deaths by cause is available directly from the Russian Fertility and Mortality database (https://ghdx.healthdata.org/series/russian-fertility-and-mortality-database-rusfmd). Data on behavioural risk factors and other oblast-level variables are available through the Rosstat data repository. All data compiled by the authors of this study are available upon request.

## Ethical clearance

None was needed as this is secondary data analysis.

## Editor note

The Lancet Group takes a neutral position with respect to territorial claims in published maps and institutional affiliations.

## Declaration of interests

We declare no competing interests.

## Appendix A Supplementary data

Figures A1–A13 and Tables A1–A10
