# Supplementary material for: Preventable mortality in the Russian Federation: a retrospective, regional level study
Source: Lancet Reg Health Eur. 2023 Apr 19;29:100631. doi: 10.1016/j.lanepe.2023.100631 (PMC10172901; doi:10.1016/j.lanepe.2023.100631)
Supplement: Figures A1–A13 and Tables A1–A10 [file mmc1.docx]

***Supplementary Materials***

Contents

[Appendix 1 – Causes of preventable and treatable mortality 2](#_Toc124862097)

[Appendix 2 – Russian nomenclature of deaths based on the ICD-10 codes 9](#_Toc124862098)

[Appendix 3 – Russian oblasts 25](#_Toc124862099)

[Appendix 4 – Adjustments made for data processing 27](#_Toc124862100)

[Appendix 5 – Additional variables used in the modelling exercise 29](#_Toc124862101)

[Appendix 6 – Additional tables and figures 30](#_Toc124862102)

# Appendix 1 – Causes of preventable and treatable mortality

**Table A1. Causes of preventable mortality.**

| **Group** | **Cause of death** | **ICD-10 codes** | **Age range**  **(years)** | **Rationale for inclusion** |
| --- | --- | --- | --- | --- |
| Infectious diseases | Intestinal diseases | A00–A09 | 0–74 | Most of these infections can be prevented by public health measures (e.g., improvements in water and food safety). |
|  | Diphtheria, Tetanus, Poliomyelitis | A35, A36, A80 | 0–74 | Most of these infections can be prevented by vaccination. |
|  | Whooping cough | A37 | 0–74 | Most of these infections can be prevented by vaccination. |
|  | Meningococcal infection | A39 | 0–74 | Most of these infections can be prevented by vaccination. |
|  | Streptococcal pneumonia; sepsis due to streptococcus  and *Haemophilus influenzae* | A40.3, A41.3 | 0–74 | Most of these infections can be prevented by vaccination. |
|  | Other *H. influenzae* infections | A49.2 | 0–74 | Most of these infections can  be prevented by vaccination |
|  | Sexually transmitted infections (other than HIV/AIDS) | A50-A60, A63, A64 | 0–74 | These infections can be prevented by public health measures. |
|  | Varicella | B01 | 0–74 | Most of these infections can  be prevented by vaccination |
|  | Measles | B05 | 0–74 | Most of these infections can be prevented by vaccination. |
|  | Rubella | B06 | 0–74 | Most of these infections can be prevented by vaccination. |
|  | Viral Hepatitis | B15–B19 | 0–74 | This condition is preventable and will not require treatment if prevented. |
|  | HIV/AIDS | B20–B24 | 0–74 | This condition is preventable and will not require treatment if prevented. |
|  | Malaria | B50-B54 | 0–74 | This condition is preventable and will not require treatment if prevented. |
|  | Hemophilus and pneumococcal  meningitis | G00.0, G00.1 | 0–74 | Most of these infections can be prevented by vaccination. |
|  | Tuberculosis*** | A15–A19, B90, J65 | 0-74 | Reduction in deaths from tuberculosis has been achieved by improved prevention (i.e., reductions in incidence) as well as earlier detection and more effective treatments (i.e., improved survival). |
| Cancer | Lip, oral cavity, and pharyngeal cancer | C00–C14 | 0–74 | This condition can be largely prevented by public health measures (e.g., programs to reduce smoking). |
|  | Esophageal cancer | C15 | 0–74 | This condition can be largely prevented by public health measures (e.g., programs to reduce smoking). |
|  | Stomach cancer | C16 | 0–74 | This condition can be largely prevented by public health measures (e.g., programs to reduce smoking and alcohol consumption and improvements in nutrition). |
|  | Liver cancer | C22 | 0–74 | This condition can be largely prevented by public health measures (e.g., programs to reduce smoking and alcohol consumption). |
|  | Lung cancer | C33–C34 | 0–74 | This condition can be largely prevented by public health measures (e.g., programs to reduce smoking). |
|  | Mesothelioma | C45 | 0–74 | This condition can be largely prevented by environmental measures (e.g., reductions in asbestos exposure). |
|  | Skin cancer (melanoma) | C43 | 0–74 | This condition can be largely prevented by environmental measures (e.g., reductions in sun exposure). |
|  | Bladder cancer | C67 | 0–74 | This condition can be largely prevented by public health measures (e.g., programs to reduce smoking). |
|  | Cervical cancer*** | C53 | 0–74 | Cervical cancer can be prevented by vaccination. Timely screening can identify pre-cancerous abnormalities that can be treated to prevent full-blown cancer. However, five-year survival after cancer detection is currently relatively high and increasing. |
| Endocrine  and metabolic  diseases | Nutritional deficiency anemia | D50–D53 | 0–74 | This condition can be largely prevented by public health measures (e.g., improved nutrition). |
|  | Diabetes mellitus*** | E10–E14 | 0–74 | Type 1 diabetes is not preventable but appropriate treatments reduce mortality. Type 2 diabetes is largely preventable (e.g., improvements in nutrition). Appropriate treatments can also reduce mortality. |
| Diseases of  the circulatory  system | Aortic aneurysm*** | I71 | 0–74 | This condition is both preventable by public health measures (similar risk factors as those identified for ischemic heart disease) and is treatable. |
|  | Hypertensive disease | I10–I13, I15 | 0–74 | This condition is preventable by public health measures (e.g., programs to reduce smoking and improvements in nutrition and physical activity) and is treatable. |
|  | Ischemic heart disease (IHD) | I20–I25 | 0–74 | Reductions in deaths associated with IHD have been achieved by improved prevention (i.e., reductions in incidence), earlier detection, and more effective treatments (i.e., improved survival). |
|  | Cerebrovascular disease (CVD) | I60–I69 | 0–74 | Reduction in deaths from CVD over the past decades in several countries has been about evenly achieved through greater prevention (reduction in incidence) and earlier detection and more effective treatment (higher survival rates). |
|  | Other diseases associated with atherosclerosis | I70, I73.9 | 0–74 | These conditions are both preventable by public health measures (e.g., improved nutrition) and treatable. |
| Diseases of  the respiratory  system | Influenza | J09–J11 | 0–74 | Most of the deaths associated with this condition can be prevented by vaccination. |
|  | Streptococcal or *H. influenzae* pneumonia | J13–J14 | 0–74 | Most of these infections can be prevented through vaccination. |
|  | Chronic lower respiratory diseases | J40–J44 | 0–74 | This condition can be largely prevented by public health measures (e.g., programs to reduce smoking). |
|  | Lung diseases due to external  agents | J60–J64, J66–J70, J82, J92 | 0–74 | These conditions can be largely prevented by environmental measures (e.g., reduced exposure to chemicals, gases, and other agents). |
| Pregnancy,  childbirth, and the  perinatal  period | Tetanus neonatorum | A33 | 0–74 | Most of these infections can be prevented by vaccination. |
|  | Obstetrical tetanus | A34 | 0–74 | Most of these infections can be prevented by vaccination |
| Congenital  malformations | Neural tube defects | Q00, Q01, Q05 | 0–74 | These conditions can largely be prevented by public health measures (e.g., improvements in maternal nutrition, notably folic acid consumption). |
| Injuries | Vehicular accidents | V01–V99 | 0–74 | Deaths can be prevented by public health measures (e.g., improved road safety). |
|  | Accidental Injuries | W00  X39, X46  X59 | 0–74 | Deaths can be prevented by public health measures (e.g., injury prevention campaigns). |
|  | Intentional self-harm | X66–X84 | 0–74 | Deaths can be prevented by public health measures (e.g., suicide prevention campaigns). |
|  | Event of undetermined intent | Y16–Y34 | 0–74 | Deaths can be prevented by public health measures (e.g., harm prevention campaigns). |
|  | Assault | X86–Y09 | 0–74 | Deaths can be prevented by public health interventions. |
|  | Specific alcohol-related disorders and  poisonings | E24.4, F10, G31.2, G62.1, G72.1, I42.6,  K29.2, K70, K85.2,  K86.0, Q86.0, R78.0,  X45, X65, Y15 | 0–74 | Deaths can be largely prevented by public health measures (e.g., alcohol control policies). |
|  | Other alcohol-  related disorders | K73, K74.0–K74.2, K74.6 | 0–74 | Deaths can be largely prevented by public health measures (e.g., alcohol control policies). |
|  | Drug-related disorders  and poisonings | F11–F16, F18–F19, X40–X44, X85, Y10–Y14 | 0–74 | Deaths can be largely prevented by public health measures (e.g., drug control policies). |
|  | Intentional  self-poisoning  by drugs | X60–X64 | 0–74 | Deaths can be largely prevented by public health measures (e.g., drug control policies). |

***50% of cases evaluated.

*Sources:* Eurostat/OECD (2019).

**Table A2. Causes of treatable mortality.**

| **Group** | **Causes of death** | **ICD-10 codes** | **Age range (years)** | **Rationale for inclusion** |
| --- | --- | --- | --- | --- |
| Infectious diseases | Tuberculosis*** | A15–A19, B90, J65 | 0–74 | Reductions in death from tuberculosis have been achieved via improved prevention (i.e., reduction in incidence), earlier detection, and more effective treatment (i.e., higher survival rates). |
|  | Scarlet fever | A38 | 0–74 | Case-fatality rates can be reduced by early detection and appropriate antibiotic treatment. |
|  | Sepsis | A40 (excl. A40.3), A41  (excl. A41.3) | 0–74 | Case-fatality rates can be reduced by improving the quality of patient care, reductions in adverse events, early detection, and appropriate antibiotic treatment. |
|  | Cellulitis | A46, L03 | 0–74 | Case-fatality rates can be reduced by early detection and appropriate antibiotic treatment. |
|  | Legionnaires’ disease | A48.1 | 0–74 | Case-fatality rates can be reduced by early detection and appropriate antibiotic treatment. |
|  | Streptococcal and enterococcal  infections | A49.1 | 0–74 | Case-fatality rates can be reduced by early detection and appropriate antibiotic treatment. |
|  | Other forms of bacterial meningitis | G00.2, G00.3, G00.8,  G00.9 | 0–74 | Case-fatality rates can be reduced by early detection and appropriate antibiotic treatment. |
|  | Bacterial meningitis due to other and unspecified causes | G03 | 0–74 | Case-fatality rates can be reduced by early detection and appropriate antibiotic treatment. |
| Cancer | Cervical cancer*** | C53 | 0–74 | Cervical cancer can be prevented by vaccination and screening to identify pre-cancerous abnormalities that can be treated. However, the five-year survival rate after the detection of a cancerous lesion is relatively high and rising. |
|  | Colorectal cancer | C18–C21 | 0–74 | Case-fatality rates have been reduced by early detection and treatment. However, the five-year survival rate after the detection of a cancerous lesion is relatively high and rising. |
|  | Breast cancer (females only) | C50 | 0–74 | Case-fatality rates have been reduced by early detection and treatment. However, the five-year survival rate after the detection of a cancerous lesion is relatively high and rising. |
|  | Uterine cancer | C54, C55 | 0–74 | Case-fatality rates have been reduced by early detection and treatment. However, the five-year survival rate after the detection of a cancerous lesion is relatively high and rising. |
|  | Testicular cancer | C62 | 0–74 | Case-fatality rates have been reduced by early detection and treatment. However, the five-year survival rate after the detection of a cancerous lesion is relatively high and rising. |
|  | Thyroid cancer | C73 | 0–74 | Case-fatality rates have been reduced by early detection and appropriate treatment. |
|  | Hodgkin's disease | C81 | 0–74 | Case-fatality rates have been reduced by early detection and appropriate treatment. |
|  | Lymphoid leukemia | C91.0, C91.1 | 0–74 | Case-fatality rates have been reduced by early detection and appropriate treatment. |
|  | Benign neoplasms | D10–D36 | 0–74 | Case-fatality rates have been reduced by early detection and appropriate treatment. |
| Endocrine  and metabolic  diseases | Diabetes mellitus*** | E10–E14 | 0–74 | Type 1 diabetes is not preventable, but appropriate treatment can reduce mortality. Whilst type 2 diabetes is largely preventable (e.g., via improvements in nutrition), appropriate treatments can also reduce mortality. |
|  | Thyroid disorders | E00–E07 | 0–74 | Case-fatality rates can be reduced by early detection and appropriate treatment. |
|  | Adrenal disorders | E24–E25 (except E24.4),  E27 | 0–74 | Case-fatality rates can be reduced by early detection and appropriate treatment. |
| Diseases of the nervous  system | Epilepsy | G40, G41 | 0–74 | Case-fatality rates can be reduced by early detection and appropriate treatment. |
| Diseases of the circulatory  system | Aortic aneurysm*** | I71 | 0–74 | This condition is preventable by public health measures (similar to the risk factors associated with ischemic heart diseases) and treatable. |
|  | Hypertensive disease*** | I10–I13, I15 | 0–74 | This condition is both preventable (e.g., programs to reduce smoking, improvements in nutrition, and physical activity) and treatable. |
|  | Ischemic heart disease (IHD)*** | I20–I25 | 0–74 | Reductions in deaths from IHD have been achieved by improved prevention (i.e., a reduction in incidence) as well as earlier detection and more effective treatments (i.e., higher survival rates). |
|  | Cerebrovascular disease (CVD)*** | I60–I69 | 0–74 | Reductions in deaths from CVD have been achieved by improved prevention (i.e., a reduction in incidence) as well as earlier detection and more effective treatments (i.e., higher survival rates). |
|  | Other sequelae of atherosclerosis*** | I00–I09 | 0–74 | Case-fatality rates can be reduced by appropriate treatment. |
|  | Venous thromboembolism | I26, I80, I82.9 | 0–74 | Most venous thrombosis occurs during hospitalisations. These cases are treatable to the extent that they are linked to the quality of care. |
| Diseases of the  respiratory  system | Upper respiratory infections | J00–J06, J30–J39 | 0–74 | Case-fatality rates can be reduced by appropriate treatment. |
|  | Pneumonia, not elsewhere  classified or organism unspecified | J12, J15, J16–J18 | 0–74 | Case-fatality rates can be reduced by early detection and appropriate antibiotic treatment. |
|  | Acute lower respiratory infections | J20–J22 | 0–74 | Case-fatality rates can be reduced by appropriate treatment. |
|  | Asthma and bronchiectasis | J45–J47 | 0–74 | Case-fatality rates can be reduced by appropriate treatment. |
|  | Adult respiratory distress syndrome | J80 | 0–74 | Case-fatality rates can be reduced by appropriate treatment. |
|  | Pulmonary edema | J81 | 0–74 | Case-fatality rates can be reduced by appropriate treatment |
|  | Abscess of lung and mediastinum;  pyothorax | J85, J86 | 0–74 | Case-fatality rates can be reduced by appropriate treatment. |
|  | Other pleural disorders | J90, J93, J94 | 0–74 | Case-fatality rates can be reduced by appropriate treatment. |
| Diseases of the digestive  system | Gastric and duodenal ulcer | K25–K28 | 0–74 | Case-fatality rates can be reduced by early detection and appropriate treatment. |
|  | Appendicitis | K35–K38 | 0–74 | Case-fatality rates can be reduced by early detection and appropriate treatment. |
|  | Abdominal hernia | K40–K46 | 0–74 | Case-fatality rates can be reduced by early detection and appropriate treatment. |
|  | Cholelithiasis and cholecystitis | K80–K81 | 0–74 | Case-fatality rates can be reduced by early detection and appropriate treatment. |
|  | Other diseases of the gallbladder or  biliary tract | K82–K83 | 0–74 | Case-fatality rates can be reduced by early detection and appropriate treatment. |
|  | Acute pancreatitis | K85.0, 1, 3, 8, 9 | 0–74 | Case-fatality rates can be reduced by early detection and appropriate treatment. |
|  | Other diseases of the pancreas | K86.1, 2, 3, 8, 9 | 0–74 | Case-fatality rates can be reduced by early detection and appropriate treatment. |
| Diseases of the  genitourinary system | Nephritis and nephrosis | N00–N07 | 0–74 | Case-fatality rates can be reduced by early detection and appropriate treatment. |
|  | Obstructive uropathy | N13, N20–N21, N35 | 0–74 | Case-fatality rates can be reduced by early detection and appropriate treatment. |
|  | Renal failure | N17–N19 | 0–74 | Case-fatality rates can be reduced by early detection and appropriate treatment. |
|  | Renal colic | N23 | 0–74 | Case-fatality rates can be reduced by early detection and appropriate treatment. |
|  | Disorders resulting from renal  tubular dysfunction | N25 | 0–74 | Case-fatality rates can be reduced by early detection and appropriate treatment. |
|  | Contracted kidney of unspecified cause;  small kidney of unknown cause | N26–N27 | 0–74 | Case-fatality rates can be reduced by early detection and appropriate treatment. |
|  | Inflammatory diseases of the genitourinary system | N34.1, N70–N73, N75.0, N75.1, N76.4,6 | 0–74 | Case-fatality rates can be reduced by early detection and appropriate treatment. |
|  | Prostatic hyperplasia | N40 | 0–74 | Case-fatality rates can be reduced by early detection and appropriate treatment. |
| Pregnancy,  childbirth, and the perinatal  period | Pregnancy, childbirth, and the perinatal period | O00–O99 | 0–74 | Effective treatment is available in most cases to prevent maternal mortality. |
|  | Conditions that originate in  the perinatal period | P00–P96 | 0–74 | Case-fatality rates can be reduced by early detection and appropriate treatment. |
| Congenital  malformations | Congenital malformations of the circulatory system (cardiac defects) | Q20–Q28 | 0–74 | These conditions can be treated by surgical procedures. |
| Adverse  events associated with medical and  surgical care | Drugs, medications, and biological substances eliciting  adverse events in therapeutic use | Y40–Y59 | 0–74 | These conditions can be treated by adjusting drug prescriptions and improving adherence. |
|  | Adverse events developing during  surgical and medical care | Y60–Y69, Y83–Y84 | 0–74 | These conditions can be treated by improving the quality of care. |
|  | Medical devices associated with  adverse events in diagnostic  and therapeutic use | Y70–Y82 | 0–74 | These conditions can be treated by improving the quality of care. |

***50% of cases evaluated.

*Sources:* Eurostat/OECD (2019).

# Appendix 2 – Russian nomenclature of deaths based on the ICD-10 codes

Appendix A3. Abbreviated Russian nomenclature of causes of death based on ICD-10 codes.

All causes of death from 1999 to 2010.

| **Code** | **Name** | **ICD-10 codes*** |
| --- | --- | --- |
| 1 | Cholera | A00 |
| 2 | Typhoid fever | A010 |
| 3 | Paratyphoid fever | A011, A012, A013, A014 |
| 4 | Other salmonella infections | A02 |
| 5 | Shigellosis | A03 |
| 6 | Other bacterial foodborne intoxications | A05 |
| 7 | Other bacterial intestinal infections including amoebiasis and other  protozoal intestinal diseases | A04, A06**-**A08 |
| 8 | Diarrhea and gastroenteritis of presumed infectious origin | A09 |
| 9 | Respiratory tuberculosis, bacteriologically and histologically  confirmed | A15 |
| 10 | Respiratory tuberculosis, not confirmed bacteriologically or  histologically | A16 |
| 11 | Tuberculosis of the nervous system | A17+ |
| 12 | Tuberculosis of the intestines, peritoneum, and mesenteric glands | A183 |
| 13 | Tuberculosis of bones and joints | A180+ |
| 14 | Tuberculosis of the genitourinary system | A181+ |
| 15 | Other forms of tuberculosis excluding late effects | A182**–**8, A19 |
| 16 | Plague | A20 |
| 17 | Anthrax | A22 |
| 18 | Brucellosis | A23 |
| 19 | Leprosy (Hansen's disease) | A30 |
| 20 | Tetanus neonatorum | A33 |
| 21 | Obstetrical tetanus | A34 |
| 22 | Other tetanus | A35 |
| 23 | Diphtheria | A36 |
| 24 | Whooping cough | A37 |
| 25 | Scarlet fever | A38 |
| 26 | Meningococcal infection | A39 |
| 27 | Septicemia | A40, A41 |
| 28 | Erysipelas | A46 |
| 29 | Other bacterial infections (excluding foodborne toxins) | A21, A24**–**A28, A31, A32, A42,  A43, A44, A48, A49 |
| 30 | Syphilis (all forms) | A50**–**A53 |
| 31 | Other predominantly sexually-transmitted diseases | A54**–**A64 |
| 32 | Relapsing fevers | A68 |
| 33 | Typhus fever | A75 |
| 34 | Spotted fever, Q fever, other rickettsioses | A77**–**A79 |
| 35 | Acute poliomyelitis | A80 |
| 36 | Rabies | A82 |
| 37 | Viral encephalitis | A83**–**A86 |
| 38 | Yellow fever | A95 |
| 39 | Other arthropod-borne viral fevers and viral haemorrhagic fevers | A90**–**A94, A96**-**A99 |
| 40 | Measles | B05 |
| 41 | Acute hepatitis A | B15 |
| 42 | Acute hepatitis B | B16 |
| 43 | Other acute and chronic viral hepatitis | B17**–**B19 |
| 44 | Human immunodeficiency virus (HIV) disease | B20**–**B24 |
| 45 | All other viral diseases | A81, A87**–**A89, B00**–**B04, B06**–**  B09, B25**–**B34 |

| 46 | Protozoal diseases (e.g., malaria) | B50**–**B54 |
| --- | --- | --- |
| 47 | Leishmaniasis | B55 |
| 48 | Trypanosomiasis | B56**–**B57 |
| 49 | Schistosomiasis (bilharziasis) | B65 |
| 50 | Echinococcosis | B67 |
| 51 | Dracunculiasis and filariasis | B72, B74 |
| 52 | Other helminthiases | B66, B68**–**B71, B73, B75**–**B83 |
| 53 | Other and unspecified infectious and parasitic diseases | A65**–**A67, A69**–**A74, B35**–**B49, B58**–**B60, B64, B85-B89, B95**–**B97, B99 |
| 54 | Sequelae of tuberculosis | B90 |
| 55 | Late effects of other infectious and parasitic diseases, including  late effects of poliomyelitis | B91**–**B94 |
| 56 | Malignant neoplasm of the lip, oral cavity, and pharynx | C00**–**C14 |
| 57 | Malignant neoplasm of the esophagus | C15 |
| 58 | Malignant neoplasm of the stomach | C16 |
| 59 | Malignant neoplasm of the small intestine | C17 |
| 60 | Malignant neoplasm of the colon | C18 |
| 61 | Malignant neoplasm of the rectum, rectosigmoid junction, and anus | C19**–**C21 |
| 62 | Malignant neoplasm of the liver and intrahepatic bile ducts | C22 |
| 63 | Malignant neoplasm of the pancreas | C25 |
| 64 | Malignant neoplasms of other and ill-defined digestive organs | C23, C24, C26 |
| 65 | Malignant neoplasm of the larynx | C32 |
| 66 | Malignant neoplasm of the trachea, bronchus, and lung | C33, C34 |
| 67 | Malignant neoplasm of other and ill-defined sites in respiratory and intrathoracic organs | C30, C31C37**–**C39 |
| 68 | Malignant neoplasm of bone and articular cartilage of limbs, other, and unspecified sites | C40, C41 |
| 69 | Malignant melanoma of the skin | C43 |
| 70 | Other malignant neoplasms of the skin | C44 |
| 71 | Mesothelioma, Kaposi's sarcoma, malignant neoplasm of peripheral nerves, the autonomic nervous system,  retroperitoneum, peritoneum, and other connective and soft tissues | C45**–**C49 |
| 72 | Malignant neoplasm of the breast | C50 |
| 73 | Malignant neoplasm of the cervix uteri | C53 |
| 74 | Malignant neoplasm of the corpus uteri and the uterus, parts unspecified | C54, C55 |
| 75 | Malignant neoplasm of the ovary | C56 |
| 76 | Malignant neoplasm of other and unspecified female genital  organs | C51, C52, C57, C58 |
| 77 | Malignant neoplasm of the prostate | C61 |
| 78 | Malignant neoplasm of other male genital organs | C60, C62, C63 |
| 79 | Malignant neoplasm of the kidney, except the renal pelvis | C64 |
| 80 | Malignant neoplasm of the bladder | C67 |
| 81 | Malignant neoplasm of other and unspecified urinary organs | C65, C66, C68 |
| 82 | Malignant neoplasm of the meninges, brain, spinal cord, cranial  Nerves, and other parts of the central nervous system | C70**–**C72 |
| 83 | Malignant neoplasms of other and independent (primary) multiple  sites | C69, C73**–**C80, C97 |
| 84 | Hodgkin's disease | C81 |
| 85 | Non-Hodgkin's lymphoma | C82**–**C85 |
| 86 | Multiple myeloma and malignant plasma cell neoplasms | C90 |
| 87 | Leukemia | C91**–**C95 |
| 88 | Other and unspecified malignant neoplasms of lymphoid,  hematopoietic, related tissue, and malignant immunoproliferative diseases | C88, C96 |

| 89 | *In situ* neoplasms, benign and unspecified neoplasms | D00**–**D48 |
| --- | --- | --- |
| 90 | Hemolytic anemias | D55**–**D59 |
| 91 | Other anemias | D50**–**D53, D60**–**D64 |
| 92 | Other diseases of the blood and blood-forming organs | D65**–**D89 |
| 93 | Diabetes mellitus | E10**–**E14 |
| 94 | Diseases of other endocrine glands | E00**–**E07, E15**–**E16, E20**–**E35 |
| 95 | Malnutrition | E40**–**E46 |
| 96 | Other nutritional and metabolic disorders | E50**–**E90 |
| 97 | Chronic alcoholism | F100**–**3, F106 (часть) F108, 9 |
| 98 | Alcoholic psychosis, encephalopathy, or dementia | F104**–**5, F106 (часть) F107 |
| 99 | Other psychoses | F00**–**F09, F20**–**F29 |
| 100 | Mental and behavioural disorders due to drug use and use of other  psychoactive substances | F11, F12, F14, F13 (часть), F15  (часть), F16 (часть), F19 (часть) |
| 101 | Mental and behavioural disorders due to the use of other psychoactive  substances | F17, F13 (часть), F15  (часть),F16 (часть),F19 (часть) |
| 102 | Mental retardation | F70**–**F79 |
| 103 | Other mental and behavioural disorders | F30**–**F69, F80**–**F99 |
| 104 | Meningitis, excluding infectious and parasitic meningitides | G00, G03 |
| 105 | Other inflammatory diseases of the central nervous system | G04, G06, G08, G09 |
| 106 | Parkinson's disease and secondary parkinsonism | G20, G21 |
| 107 | Alzheimer's disease | G30 |
| 108 | Multiple sclerosis | G35 |
| 109 | Epilepsy | G40, G41 |
| 110 | Infantile cerebral palsy | G80 |
| 111 | Other diseases of the nervous system | G10**–**G12, G23**–**G25, G31, G36,  G37, G43**–**G45, G47, G50**–**G72, G81**–**G98 |
| 112 | Diseases of the eye and adnexa | H00**–**H59 |
| 113 | Otitis media | H65**–**H66 |
| 114 | Other diseases of the ear and mastoid process | H60**–**H62, H70**–**H95 |
| 115 | Acute rheumatic fever | I00**–**I02 |
| 116 | Chronic rheumatic heart disease | I05**–**I09 |
| 117 | Hypertensive heart disease | I11 |
| 118 | Hypertensive renal disease | I12 |
| 119 | Hypertensive heart and renal disease | I13 |
| 120 | Other and unspecified hypertensive disease | I10, I15 |
| 121 | Acute myocardial infarction including certain immediate complications following acute myocardial infarction | I21, I23 |
| 123 | Delayed myocardial infarction | I22 |
| 125 | Atherosclerotic heart disease | I251 |
| 127 | Other forms of chronic ischemic heart disease | I252**–**9 |
| 129 | Other forms of acute ischemic heart disease | I20, I241**–**9 |
| 131 | Pulmonary heart disease and diseases of pulmonary circulation | I26**–**I28 |
| 132 | Other forms of heart disease | I30**–**I51 |
| 133 | Subarachnoid haemorrhage | I60 |
| 135 | Intracerebral haemorrhage and other nontraumatic intracranial  haemorrhage | I61**–**I62 |
| 137 | Cerebral infarction | I63 |
| 139 | Stroke, not specified as haemorrhage or infarction | I64 |
| 141 | Other cerebrovascular diseases | I67**–**I69 |
| 143 | Atherosclerosis | I70 |
| 144 | Other diseases of arteries, arterioles, and capillaries | I71**–**I79 |
| 145 | Phlebitis and thrombophlebitis, venous embolism, and thrombosis | I80**–**I82 |
| 146 | Other disorders of veins and lymphatic vessels | I83**–**I89 |
| 147 | Other and unspecified diseases of the circulatory system | I95**–**I99 |
| 148 | Acute upper respiratory infection | J00**–**J01, J028**–**9, J03**–**J06 |

| 149 | Streptococcal pharyngitis | J020 |
| --- | --- | --- |
| 150 | Influenza | J10**–**J11 |
| 151 | Viral pneumonia, not elsewhere classified | J12 |
| 152 | Bacterial pneumonia | J13**–**J15 |
| 153 | Pneumonia due to other infectious organisms, not elsewhere  classified | J16 |
| 154 | Pneumonia, organism unspecified | J18 |
| 155 | Acute lower respiratory infection | J20**–**J22 |
| 156 | Bronchitis, not specified as acute or chronic | J40 |
| 157 | Emphysema | J43 |
| 158 | Other chronic obstructive pulmonary disease | J42, J41, J44 |
| 159 | Asthma | J45**–**J46 |
| 160 | Bronchiectasis | J47 |
| 161 | Pneumoconiosis and other lung diseases due to exogenous agents | J60**–**J70 |
| 162 | Other respiratory diseases principally affecting the interstitium | J80**–**J84 |
| 163 | Suppurative and necrotic conditions of the lower respiratory tract | J85**–**J86 |
| 164 | Other diseases of the respiratory system | J30**–**J39, J90**–**J99 |
| 165 | Gastric ulcer | K25 |
| 166 | Duodenal ulcer | K26 |
| 167 | Peptic ulcer, site unspecified | K27 |
| 168 | Gastritis and duodenitis | K29 |
| 169 | Diseases of appendix | K35**–**K38 |
| 170 | Hernia | K40**–**K46 |
| 171 | Noninfectious enteritis and colitis | K50**–**K52 |
| 172 | Paralytic ileus and intestinal obstruction without hernia | K56 |
| 173 | Alcoholic liver disease (alcoholic cirrhosis, hepatitis, fibrosis) | K70 |
| 174 | Fibrosis and cirrhosis of the liver | K74 |
| 175 | Other diseases of the liver | K71**–**K73, K75**–**K76 |
| 176 | Cholelithiasis | K80 |
| 177 | Cholecystitis | K81 |
| 178 | Acute pancreatitis and other diseases of the pancreas | K85**–**K86 |
| 179 | Other diseases of the digestive system | K00**–**K14, K20**–**K24, R28, K30**–** K31, K55, K57**–**K66, K82, K83,  K90**–**K93 |
| 180 | Diseases of the skin and subcutaneous tissue | L00**–**L98 |
| 181 | Rheumatoid arthritis | M05**–**M06 |
| 182 | Systemic lupus erythematosus | M32 |
| 183 | Osteomyelitis and periostitis | M86 |
| 184 | Other diseases of the musculoskeletal system and connective  tissue | M00**–**M03, M08, M10**–**M31, M33**–**M85, M87**–**M99 |
| 185 | Acute and rapidly progressive nephritic syndrome (acute nephritis, glomerulonephritis, glomerular disease) | N00**–**N01 |
| 186 | Chronic nephritic syndrome (chronic glomerular disease,  glomerulonephritis, nephritis) | N03 |
| 187 | Other glomerular disorders (isolated nephritic syndrome, hereditary nephropathy, nephrotic syndrome unspecified) | N02, N04**–**N08 |
| 188 | Acute tubulointerstitial nephritis (acute infectious interstitial  nephritis, pyelitis, pyelonephritis) | N10 |
| 189 | Other renal tubulointerstitial diseases | N11**–**N15 |
| 190 | Urolithiasis (calculus of urinary tract) | N20**–**N23 |
| 191 | Other diseases of the urinary system | N17**–**N19, N25**–**N39 |
| 192 | Hyperplasia of the prostate | N40 |
| 193 | Other diseases of genital organs | N41**–**N99 |
| 194 | Ectopic pregnancy | O00 |
| 195 | Legally-induced abortion (medical abortion, failed attempted  abortion) | O04, O07 |

| 196 | Abortion in-hospital and unspecified | O01**–**O03, O05, O06, O08 |
| --- | --- | --- |
| 197 | Toxemia in pregnancy | O10, O11, O13**–**O15, O21 |
| 198 | Haemorrhage due to placenta previa and separation of the placenta | O441, O45, O20 |
| 199 | Antepartum and postpartum haemorrhage | O46, O720**–**3, O670, 8, 9 |
| 200 | Complications of anesthesia during pregnancy, labor, delivery, and the perinatal period | O29, O74, O89 |
| 201 | Septicemia in childbirth and the perinatal period | O85, O860O883, O911 |
| 202 | Obstetric embolism | O880-2, O888 |
| 203 | Other direct obstetric causes | O12, O22**–**O28, O30**–**O43, O440, O47, O48, O60**–**O66, O68**–**O71, O73, O75, O80**–**O84, O861**–**4,8  O87, O90, O912, O92 |
| 204 | Indirect obstetric causes | O98, O99 |
| 205 | Other complications of pregnancy, childbirth, and the perinatal period (late maternal death more than 42 days after delivery) | O95**–**O97 |
| 206 | Birth trauma | P10**–**P15 |
| 207 | Intrauterine hypoxia and birth asphyxia | P20**–**P21 |
| 208 | Respiratory distress of the newborn | P22 |
| 209 | Congenital pneumonia | P23 |
| 210 | Other respiratory disorders specific to the perinatal period | P24**–**P28 |
| 211 | Bacterial sepsis of the newborn | P36 |
| 212 | Omphalitis of the newborn with or without mild haemorrhage | P38 |
| 213 | Hemorrhagic disorders of newborn | P50**–**P54 |
| 214 | Hemolytic disease and other causes of perinatal jaundice | P55**–**P61 |
| 215 | Transitory endocrine and metabolic disorders specific to the newborn | P70**–**P74 |
| 216 | Other conditions originating in the perinatal period | P29, P35, P37, P39, P75**–**P96,  P05**–**P08 |
| 217 | Spina bifida and congenital hydrocephalus | Q03, Q05 |
| 218 | Other congenital anomalies of the central nervous system | Q00**–**Q02, Q04, Q06**–**Q07 |
| 219 | Congenital anomalies of the heart | Q20**–**Q24 |
| 220 | Other congenital anomalies of the circulatory system | Q25**–**Q28 |
| 221 | Congenital malformations of the respiratory system | Q30**–**Q34 |
| 222 | Congenital anomalies of the digestive system | Q35**–**Q45 |
| 223 | Congenital malformations of the urinary system | Q60**–**Q64 |
| 224 | Down syndrome and other chromosomal abnormalities, not  elsewhere classified | Q90**–**Q99 |
| 225 | Other congenital malformations, deformations, and chromosomal  abnormalities | Q10**–**Q18, Q50**–**Q56, Q65**–**Q89 |
| 226 | Senility | R54 |
| 227 | Sudden infant death syndrome | R95 |
| 228 | Other ill-defined and unspecified causes of mortality | R00**–**R53, R55**–**R94, R96-**–**R99 |
| 229 | Injuries to the head | S00**–**S09 |
| 230 | Injuries to the neck | S10**–**S19 |
| 231 | Injuries to the thorax | S20**–**S29 |
| 232 | Injuries to the abdomen, lower back, lumbar spine, and pelvis | S30**–**S39 |
| 233 | Injuries to the limbs | S40**–**S99 |
| 234 | Effects of foreign body entering through a natural orifice | T15**–**T19 |
| 235 | Burns and corrosions | T20**–**T32 |
| 236 | Frostbite | T33**–**T35 |
| 237 | Poisoning by drugs, medications, and biological substances; toxic  effects of substances of chiefly non-medicinal sources | T36**–**T65 |
| 238 | Other injuries, poisonings, and consequences of external causes | T00**–**T14, T66**–**T98 |
| 239 | Pedestrian injured in transport accident | V021**–**9;V031**–**9;V041**–**9. V092, V093 |
| 240 | Car occupant injured in transport accident | V40**–**V49 |

| 241 | Other and unspecified vehicular accidents | Отсальные из V00**–**V99 |
| --- | --- | --- |
| 242 | Accidental fall | W00**–**W19 |
| 243 | Accidental drowning and submersion | W65**–**W74 |
| 244 | Accidental inhalation and ingestion resulting in obstruction of  the respiratory tract, foreign bodies entering other orifices | W75**–**W84 |
| 245 | Accidents caused by electric current, radiation, and extreme  external temperature or atmospheric pressure | W85**–**W99 |
| 246 | Accidents caused by fire or smoke | X00**–**X09 |
| 247 | Accidental poisoning by and exposure to alcohol | X45 |
| 248 | Accidental poisoning by and exposure to other poisonous  substances | X40**–**X44, X46**–**X49 |
| 249 | Suicide and self-inflicted injury | X60**–**X84 |
| 250 | Homicide and injury purposely inflicted on other persons, including legal execution | X85**–**V09 |
| 251 | Injury with undetermined intentions (whether accidentally or  purposely inflicted) | Y10**–**Y34 |
| 252 | Injury due to the operations of war | Y36 |
| 253 | Adverse events experienced by patients during therapeutic or surgical/medical care | Y40**–**Y84 |
| 254 | Other and unspecified accidents, late effects of external causes | W20**–**W31, W35**–**W64, X10**–**V39, X50**–**V59, Y35, Y85**–**Y89 |
| 255 | Accidents associated with firearm use | W32**–**W34 |
| 256 | Terrorism | Z654 |

Additions and changes made to the list in 2006.

| **Code** | **Name** | **ICD-10 codes*** |
| --- | --- | --- |
| 241 | Injured whilst an occupant of another vehicle involved in a vehicular accident | V123**–**9;V133-9;V143**–**9;V194**–**  6;V803**–**5;V811;V821;V892 |
| 272 | Injured as a pedestrian in a collision with a motor vehicle in a non-traffic related accident. | V020;V030;V040;V090**–**1; |
| 273 | Injury sustained in a collision with a motor vehicle, non-traffic related. | V120**–**2;V130**–**2;V140- 2;V190–2;V200-3;V210-3;V220-  2;V230-2;V240-2;V250-3;V260-  3;V270-3;V280-2;V290-3;V300-  4;V310-4;V320-3;V330-3;V340-  3;V350-4;V360-4;V370-4;V380-  3;V390-3;V400-4;V410-4;V420-  3;V430-3;V440-3;V450-4;V460-  4;V470-4;V480-3;V490-3;V500-  4;V510-4;V520-3;V530-3;V540-  3;V550-4;V560-4;V570-4;V580-  3;V590-3;V600-4;V610-4;V620-  3;V630-3;V640-3;V650-4;V660-  4;V670-4;V680-3;V690-3;V700-  4;V710-4;V720-3;V730-3;V740-  3;V750-4;V760-4;V770-4;V780-  3;V790-3;V810;V820;V844-  9;V854-9;V864-9;V880-8;V890; |
| 274 | Other vehicular accidents associated with other or unspecified  motor vehicles | Отсальные из V00-V99 |
| 275 | Degeneration of the nervous system due to alcohol | G312 |
| 276 | Alcoholic cardiomyopathy | I426 |
| 277 | Alcohol-induced chronic pancreatitis | K860 |
| 278 | Accidental poisoning by and exposure to narcotics and  psychodysleptics (e.g., hallucinogens), not elsewhere classified | X42 |

All causes of death; list used since 2011

| **Code** | **Name** | **ICD-10 codes*** |
| --- | --- | --- |
| 1 | Cholera | A00 |
| 2 | Typhoid fever | A010 |
| 3 | Paratyphoid fever | A011, A012, A013, A014 |
| 4 | Other salmonella infections | A02 |
| 5 | Shigellosis | A03 |
| 6 | Other bacterial foodborne toxins/illnesses  excluding salmonellosis | A05 |
| 7 | Other bacterial intestinal infections including amoebiasis and other  protozoal intestinal diseases | A04, A06-A08 |
| 8 | Diarrhea and gastroenteritis of presumed infectious origin | A09 |
| 9 | Respiratory tuberculosis, bacteriologically and histologically  confirmed | A15 |
| 10 | Respiratory tuberculosis, not confirmed bacteriologically or  histologically | A16 |
| 11 | Other tuberculosis including late effects | A17-A19, B90 |
| 12 | Plague | A20 |
| 13 | Anthrax | A22 |
| 14 | Brucellosis | A23 |
| 15 | Leprosy (Hansen's disease) | A30 |
| 16 | Tetanus neonatorum | A33 |
| 17 | Obstetrical tetanus | A34 |
| 18 | Other tetanus | A35 |
| 19 | Diphtheria | A36 |
| 20 | Whooping cough | A37 |
| 21 | Scarlet fever | A38 |
| 22 | Meningococcal infection | A39 |
| 23 | Septicemia | A40, A41 |
| 24 | Erysipelas | A46 |
| 25 | Other bacterial infections excluding foodborne illnesses | A21, A24-  A28, A31, A32, A42, A43, A44, A48, A49 |
| 26 | Syphilis (all forms) | A50-A53 |
| 27 | Other predominantly sexually-transmitted diseases | A54-A64 |
| 28 | Relapsing fevers (relapsing typhoid fever) | A68 |
| 29 | Typhus fever | A75 |
| 30 | Other rickettsioses | A77-A79 |
| 31 | Acute poliomyelitis | A80 |
| 32 | Rabies | A82 |
| 33 | Viral encephalitis | A83-A86 |
| 34 | Yellow fever | A95 |
| 35 | Other arthropod-borne viral fevers and viral hemorrhagic fevers | A90-A94, A96-A99 |
| 36 | Measles | B05 |
| 37 | Acute hepatitis A | B15 |
| 38 | Acute hepatitis B | B16 |
| 39 | Acute viral hepatitis C | B171 |
| 40 | Chronic viral hepatitis B | B180-B181 |
| 41 | Chronic viral hepatitis C | B182 |
| 42 | Other acute and chronic viral hepatitis | B172-9, B183-B189, B19 |
| 43 | Human immunodeficiency virus (HIV) disease | B20-B24 |
| 44 | All other viral diseases | A81, A87-A89, B00-B04, B06-  B09, B25-B34 |
| 45 | Protozoal diseases (e.g., malaria) | B50-B54 |
| 46 | Leishmaniasis | B55 |

| 47 | Trypanosomiasis | B56-B57 |
| --- | --- | --- |
| 48 | Schistosomiasis (bilharziasis) | B65 |
| 49 | Echinococcosis | B67 |
| 50 | Dracunculiasis and filariasis | B72,B74 |
| 51 | Other helminthiases | B66, B68-B71, B73, B75-B83 |
| 52 | Other and unspecified infectious and parasitic diseases | A65-A67, A69-A74, B35-B49, B58-B60, B64, B85-B89, B95-B97, B99 |
| 53 | Late effects of other infectious and parasitic diseases, including  late effects of poliomyelitis | B91-B94 |
| 54 | Malignant neoplasm of the lip, oral cavity, and pharynx | C00-C14 |
| 55 | Malignant neoplasm of the esophagus | C15 |
| 56 | Malignant neoplasm of the stomach | C16 |
| 57 | Malignant neoplasm of the small intestine, including duodenum | C17 |
| 58 | Malignant neoplasm of the colon | C18 |
| 59 | Malignant neoplasm of the rectum, rectosigmoid junction, and anus | C19-C21 |
| 60 | Malignant neoplasm of the liver and intrahepatic bile ducts | C22 |
| 61 | Malignant neoplasm of the pancreas | C25 |
| 62 | Malignant neoplasm of other and ill-defined digestive organs | C23, C24, C26 |
| 63 | Malignant neoplasm of the larynx | C32 |
| 64 | Malignant neoplasm of the trachea, bronchus, and lung | C33, C34 |
| 65 | Malignant neoplasm of other and ill-defined sites respiratory and  intrathoracic organs | C30, C31, C37-C39 |
| 66 | Malignant neoplasm of bone and articular cartilage of the limbs and other unspecified sites | C40 ,C41 |
| 67 | Malignant melanoma of the skin | C43 |
| 68 | Other malignant neoplasms of the skin | C44 |
| 69 | Mesothelioma, Kaposi's sarcoma, malignant neoplasm of peripheral nerves, the autonomic nervous system,  retroperitoneum, peritoneum, and other connective and soft tissue | C45-C49 |
| 70 | Malignant neoplasm of the breast | C50 |
| 71 | Malignant neoplasm of the cervix uteri | C53 |
| 72 | Malignant neoplasm of the corpus uteri and uterus, part unspecified | C54, C55 |
| 73 | Malignant neoplasm of the ovary | C56 |
| 74 | Malignant neoplasm of other and unspecified female genital  organs | C51, C52, C57, C58 |
| 75 | Malignant neoplasm of the prostate | C61 |
| 76 | Malignant neoplasm of other male genital organs | C60, C62, C63 |
| 77 | Malignant neoplasm of the kidney, except the renal pelvis | C64 |
| 78 | Malignant neoplasm of the bladder | C67 |
| 79 | Malignant neoplasm of other and unspecified urinary organs | C65, C66, C68 |
| 80 | Malignant neoplasm of the meninges, brain, spinal cord, cranial  nerves and other parts of the central nervous system | C70-C72 |
| 81 | Malignant neoplasm of the thyroid gland | C73 |
| 82 | Hodgkin's disease | C81 |
| 83 | Non-Hodgkin's lymphoma | C82-C85 |
| 84 | Multiple myeloma and malignant plasma cell neoplasms | C90 |
| 85 | Leukemia | C91-C95 |
| 86 | Malignant neoplasms of other and independent (primary) multiple  sites | C69, C74-C80, C88, C96, C97 |
| 87 | *In situ* neoplasms, benign and unspecified neoplasms | D00-D48 |
| 88 | Hemolytic anemias | D55-D59 |
| 89 | Other anemias | D50-D53, D60-D64 |
| 90 | Other diseases of the blood and blood-forming organs, including  abnormalities involving immune mechanisms | D65-D89 |
| 91 | Insulin-dependent diabetes mellitus | E10 |

| 92 | Non-insulin-dependent diabetes mellitus | E11 |
| --- | --- | --- |
| 93 | Other forms of diabetes mellitus | E12-E14 |
| 94 | Malnutrition | E40-E46 |
| 95 | Diseases of other endocrine glands, other nutritional and  metabolic disorders | E00-E07, E15-E16, E20-E35, E50-  E90 |
| 96 | Mental and behavioural disorders due to the use of alcohol, acute  intoxication | F100 |
| 97 | Mental and behavioural disorders due to the use of alcohol, harmful  use | F101 |
| 98 | Mental and behavioural disorders due to the use of alcohol,  dependence syndrome | F102 |
| 99 | Other mental and behavioural disorders due to the use of alcohol | F103, F106, F108, F109 |
| 100 | Alcoholic psychosis, encephalopathy, dementia | F104-5, F106(чACTь), F107 |
| 101 | Other psychoses | F00-F09, F20-F29 |
| 102 | Mental and behavioural disorders due to drug use and use of other  psychoactive substances | F11, F12, F14, F13(чACTь), F15(чACTь), F16(чACTь), F19(чACTь) |
| 103 | Mental and behavioural disorders due to the use of other psychoactive  substances | F17,F13(чACTь),F15(чACTь),F  16(чACTь), F19(чACTь) |
| 104 | Mental retardation |  |
| 105 | Other mental and behavioural disorders | F30-F69, F70-F79, F80-F99 |
| 106 | Meningitis, excluding infectious and parasitic meningitides | G00, G03 |
| 107 | Other inflammatory diseases of the central nervous system | G04, G06, G08, G09 |
| 108 | Parkinson's disease and secondary parkinsonism | G20, G21 |
| 109 | Alzheimer's disease | G30 |
| 110 | Multiple sclerosis | G35 |
| 111 | Epilepsy | G40, G41 |
| 112 | Infantile cerebral palsy | G80 |
| 113 | Degeneration of the nervous system due to alcohol | G312 |
| 114 | Toxic encephalopathy | G92 |
| 115 | Alcoholic polyneuropathy | G621 |
| 116 | Alcoholic myopathy | G721 |
| 117 | Other disorders of the nervous system | G10-G12, G14, G23- G25, G310, 1, 8, 9, G36, G37, G43- G45, G47, G50-G61, G620,2- 9, G63-G71, G720, 2-9, G81-  G91, G93-G98 |
| 118 | Diseases of the eye and adnexa | H00-H57, H59 |
| 119 | Otitis media | H65-H66 |
| 120 | Other diseases of the ear and mastoid process | H60-H62, H70-H93, H95 |
| 121 | Acute rheumatic fever | I00-I02 |
| 122 | Chronic rheumatic heart diseases | I05-I09 |
| 123 | Hypertensive heart disease | I11 |
| 124 | Hypertensive renal disease | I12 |
| 125 | Hypertensive heart and renal disease | I13 |
| 126 | Other and unspecified hypertensive disease | I10 |
| 127 | Acute myocardial infarction including certain immediate complications following acute myocardial infarction | I21, I23 |
| 128 | Delayed myocardial infarction | I22 |
| 129 | Atherosclerotic heart disease | I251 |
| 130 | Atherosclerotic cardiovascular disease | I250 |
| 131 | Chronic ischemic heart disease, unspecified | I259 |
| 132 | Other forms of chronic ischemic heart diseases | I252-6,8 |
| 133 | Other forms of acute ischemic heart diseases | I20, I241-9 |
| 134 | Pulmonary heart disease and diseases of pulmonary circulation | I26-I28 |
| 135 | Alcoholic cardiomyopathy | I426 |
| 136 | Cardiomyopathy, unspecified | I429 |

| 137 | Myocardial degeneration | I515 |
| --- | --- | --- |
| 138 | Heart failure, unspecified | I509 |
| 139 | Sudden cardiac death, so described | I461 |
| 140 | Other forms of heart disease | I30-I41, I420-5,7,8, I43- I45, I460,9, I47-I49, I500, 1, I510-  4, I516-9 |
| 141 | Subarachnoid haemorrhage | I60 |
| 142 | Intracerebral haemorrhage and other nontraumatic intracranial  haemorrhages | I61-I62 |
| 143 | Cerebral infarction | I63 |
| 144 | Stroke, not specified as haemorrhage or infarction | I64 |
| 145 | Cerebral atherosclerosis | I672 |
| 146 | Hypertensive encephalopathy | I674 |
| 147 | Other specified cerebrovascular diseases | I678 |
| 148 | Cerebrovascular disease, unspecified | I679 |
| 149 | Sequelae of cerebrovascular disease | I69 |
| 150 | Other cerebrovascular diseases | I670,1,3,5-7,I68 |
| 151 | Atherosclerosis | I70 |
| 152 | Other diseases of arteries, arterioles, and capillaries | I71-I79 |
| 153 | Phlebitis and thrombophlebitis, venous embolism, and thrombosis | I80-I82 |
| 154 | Other disorders of veins and lymphatic vessels | I83-I89 |
| 155 | Other and unspecified diseases of the circulatory system | I95-I99 |
| 156 | Acute upper respiratory infections | J00-J01, J028-9, J03-J06 |
| 157 | Streptococcal pharyngitis | J020 |
| 158 | Influenza | J09, J10-J11 |
| 159 | Viral pneumonia, not elsewhere classified | J12 |
| 160 | Bacterial pneumonia | J13-J15 |
| 161 | Pneumonia due to other infectious organisms, not elsewhere  classified | J16 |
| 162 | Pneumonia, organism unspecified | J18 |
| 163 | Acute lower respiratory infections (acute bronchitis and  bronchiolitis) | J20-J22 |
| 164 | Bronchitis, not specified as acute or chronic | J40 |
| 165 | Emphysema | J43 |
| 166 | Other chronic obstructive pulmonary disease | J42,J41,J44 |
| 167 | Asthma | J45-J46 |
| 168 | Bronchiectasis | J47 |
| 169 | Pneumoconiosis and other lung diseases due to external agents | J60-J70 |
| 170 | Other respiratory diseases principally affecting the interstitium | J80-J84 |
| 171 | Suppurative and necrotic conditions of the lower respiratory tract | J85-J86 |
| 172 | Other diseases of the respiratory system | J30-J39, J90-J99 |
| 173 | Gastric ulcer | K25 |
| 174 | Duodenal ulcer | K26 |
| 175 | Peptic ulcer, site unspecified | K27 |
| 176 | Alcoholic gastritis | K292 |
| 177 | Other gastritis and duodenitis | K290,1,3-9 |
| 178 | Diseases of appendix | K35-K38 |
| 179 | Hernia | K40-K46 |
| 180 | Noninfective enteritis and colitis | K50-K52 |
| 181 | Paralytic ileus and intestinal obstruction without hernia | K56 |
| 182 | Alcoholic liver disease (alcoholic fibrosis, hepatitis, cirrhosis) | K70 |
| 183 | Fibrosis and cirrhosis of the liver (except alcoholic) | K74 |
| 184 | Other diseases of the liver | K71-K73, K75-K76 |
| 185 | Cholelithiasis | K80 |
| 186 | Cholecystitis | K81 |
| 187 | Alcohol-induced chronic pancreatitis | K860 |

| 188 | Acute pancreatitis and other diseases of the pancreas | K85, K861-9 |
| --- | --- | --- |
| 189 | Vascular disorders of the intestine | K55 |
| 190 | Peritonitis | K65 |
| 191 | Other diseases of the digestive system | K00-K14,K20-K24,K28,K30- K31,K57-K64,K66,K82,K83,K90-  K93 |
| 192 | Infections of the skin and subcutaneous tissue | L00-L08 |
| 193 | Noninfective disorders of the skin and subcutaneous tissue | L10-L98 |
| 194 | Rheumatoid arthritis | M05-M06 |
| 195 | Systemic lupus erythematosus | M32 |
| 196 | Osteomyelitis | M86 |
| 197 | Other diseases of the musculoskeletal system and connective  tissue | M00-M03,M08,M10-M31,M33-  M85, M87-M99 |
| 198 | Acute and rapidly progressive nephritic syndrome (acute  nephritis, glomerulonephritis, glomerular disease) | N00-N01 |
| 199 | Chronic nephritic syndrome (chronic glomerular disease,  glomerulonephritis, nephritis) | N03 |
| 200 | Other glomerular diseases (isolated nephritic syndrome,  hereditary nephropathy, nephrotic syndrome unspecified) | N02, N04, N05-N08 |
| 201 | Acute tubulointerstitial nephritis (acute infectious interstitial  nephritis, pyelitis, pyelonephritis) | N10 |
| 202 | Other renal tubulointerstitial diseases | N11-N15 |
| 203 | Urolithiasis (calculus of urinary tract) | N20-N23 |
| 204 | Other diseases of the urinary system | N17-N19, N25-N39 |
| 205 | Hyperplasia of prostate | N40 |
| 206 | Other diseases of genital organs | N41-N99 |
| 207 | Ectopic pregnancy | O00 |
| 208 | Legally induced abortion (medical abortion and failed attempted  abortion) | O04, O07 |
| 209 | Abortion in-hospital and unspecified | O01-O03, O05, O06, O08 |
| 210 | Toxemia in pregnancy | O10-O16 |
| 211 | Haemorrhage due to placenta praevia and separation of the placenta | O441,O45,O20 |
| 212 | Antepartum and postpartum haemorrhage | O46,O720-3,O670,8,9 |
| 213 | Complications of anesthesia during pregnancy, labor and  delivery, the perinatal period | O29, O74, O89 |
| 214 | Septicemia in childbirth and the perinatal period | O85,O860,O883,O910,1 |
| 215 | Obstetric embolism | O880-2, O888 |
| 216 | Rupture of the uterus before onset or during labor | O710-O711 |
| 217 | Other direct obstetric causes | O21-O28,O30- O43,O440,O47,O48,O60- O66,O68-O70,O712- 9,O73,O75,O80-O84,O861-  4,8O87,O90,O912,O92 |
| 218 | Indirect obstetric causes | O98, O99 |
| 219 | Other complications of pregnancy, childbirth, and the perinatal period | O95, O97 |
| 220 | Death from any obstetric cause occurring more than 42 days but  less than one year after delivery | O96 |
| 221 | Birth trauma | P10-P15 |
| 222 | Intrauterine hypoxia and birth asphyxia | P20-P21 |
| 223 | Respiratory distress of the newborn | P22 |
| 224 | Congenital pneumonia | P23 |
| 225 | Other respiratory disorders specific to the perinatal period | P24-P28 |
| 226 | Bacterial sepsis of newborn | P36 |
| 227 | Omphalitis of the newborn with or without mild haemorrhage | P38 |

| 228 | Haemorrhagic disorders of newborn | P50-P54 |
| --- | --- | --- |
| 229 | Haemolytic disease and other forms of perinatal jaundice | P55-P61 |
| 230 | Transitory endocrine and metabolic disorders specific to the fetus and the newborn | P70-P74 |
| 231 | Other conditions originating in the perinatal period | P29,P35,P37,P39,P75-P96,P05-  P08 |
| 232 | Spina bifida and congenital hydrocephalus | Q03, Q05 |
| 233 | Other congenital anomalies of the central nervous system | Q00-Q02, Q04, Q06-Q07 |
| 234 | Congenital anomalies of the heart | Q20-Q24 |
| 235 | Other congenital anomalies of the circulatory system | Q25-Q28 |
| 236 | Congenital malformations of the respiratory system | Q30-Q34 |
| 237 | Congenital anomalies of the digestive system | Q35-Q45 |
| 238 | Congenital malformations of the urinary system | Q60-Q64 |
| 239 | Down syndrome and other chromosomal abnormalities, not  elsewhere classified | Q90-Q99 |
| 240 | Fetal alcohol syndrome (dysmorphic) | Q860 |
| 241 | Other congenital malformations, deformations, and chromosomal  abnormalities | Q10-Q18, Q50-Q56,Q65-  Q85, Q861-8, Q87-Q89 |
| 242 | Senility | R54 |
| 243 | Sudden infant death syndrome | R95 |
| 244 | Other sudden death, cause unknown and unattended death | R96-R99 |
| 245 | Other ill-defined and unspecified causes of mortality | R00-R53, R55-R94, |
| 246 | Injuries to the head | S00-S09 |
| 247 | Injuries to the neck | S10-S19 |
| 248 | Injuries to the thorax | S20-S29 |
| 249 | Injuries to the abdomen, lower back, lumbar spine, and pelvis | S30-S39 |
| 250 | Limb injuries | S40-S99 |
| 251 | Consequences of a foreign body entering through natural  orifice | T15-T19 |
| 252 | Thermal and chemical burns | T20-T32 |
| 253 | Frostbite | T33-T35 |
| 254 | Adverse reactions to chemical and biological substances  (i.e., poisoning) | T36-T65 |
| 255 | All other neutochnen nye-injury and the consequences of external  factors | T00-T14, T66-T98 |
| 256 | Pedestrian injured in road traffic (excluding rail) | V011,V021,V031,V041,V092-  3,V832,V842,V852,V862 |
| 257 | Cyclist (any), motorcyclist (any), or any person in a three-wheeled vehicle injured in road traffic | V103-9,V113-9,V123-9,V133- 9,V143-9,V153-9,V163-9,V173-  9,V183-9,V194-9,V203-9,V213-  9,V223-9,V233-9,V243-9,V253-  9,V263-9,V273-9,V283-9,V294-  9,V304-9,V314-9,V324-9,V334-  9,V344-9,V354-9,V364-9,V374-  9,V384-9,V394-9 |
| 258 | A person found in the vehicle who was injured in road traffic | V404-9,V414-9,V424-9,V434- 9,V444-9,V454-9,V464-9,V474-  9,V484-9,V494-9,V504-9,V514-  9,V524-9,V534-9,V544-9,V554-  9,V564-9,V574-9,V584-9,V594-  9,V604-9,V614-9,V624-9,V634-  9,V644-9,V654-9,V664-9,V674-  9,V684-9,V694-9,V704-9,V714-  9,V724-9,V734-9,V744-9,V754-  9,V764-9,V774-9,V784-9,V794-9 |

| 259 | Pedestrian injured in a moving vehicle (excluding rail) | V010,V020,V030,V040,V090,V0  91,V837,V847,V857,V867 |
| --- | --- | --- |
| 260 | Cyclist (any), motorcyclist (any), or any person in a three-wheeled vehicle who was injured in a moving vehicle. | V100-2,V110-2,V120-2,V130- 2,V140-2,V150-2,V160-2,V170-  2,V180-2,V190-3,V200-2,V210-  2,V220-2,V230-2,V240-2,V250-  2,V260-2,V270-2,V280-2,V290-  3,V300-3,V310-3,V320-3,V330-  3,V340-3,V350-3,V360-3,V370-  3,V380-3,V390-3 |
| 261 | A person found in a vehicle affected by the-road case | V400-3,V410-3,V420-3,V430- 3,V440-3,V450-3,V460-3,V470-  3,V480-3,V490-3,V500-3,V510-  3,V520-3,V530-3,V540-3,V550-  3,V560-3,V570-3,V580-3,V590-  3,V600-3,V610-3,V620-3,V630-  3,V640-3,V650-3,V660-3,V670-  3,V680-3,V690-3,V700-3,V710-  3,V720-3,V730-3,V740-3,V750-  3,V760-3,V770-3,V780-3,V790-3 |
| 262 | Pedestrian injured in a collision with a train or other railway  vehicle | V05 |
| 263 | Pedestrian injured in an unspecified vehicular accident (excluding rail) | V019,V029,V039,V049,V099 |
| 264 | Immersion in water and drowning in an accident involving a watercraft | V90 |
| 265 | Immersion in water and drowning related to a watercraft but not  associated with an accident | V92 |
| 266 | Other and unspecified accidents involving water transport | V91, V93, V94 |
| 267 | Accidents in air transportation and space travel | V95-V97 |
| 268 | Other and unspecified transportation accidents | V06,V80-V82,V830-1,V833- 6,V839,V840-1,V84-  6,V849,V850-1,V853-  6,V859,V860-1,V863-  6,V869,V87-V89,V98-V99 |
| 269 | Fall on the same level | W00-W09, W18 |
| 270 | Fall from one level to another | W10-W17 |
| 271 | Unspecified fall | W19 |
| 272 | Accidental drowning and submersion in an artificial reservoir | W65, W67 |
| 273 | Accidental drowning and submersion due to fall in the artificial  pond | W66, W68 |
| 274 | Accidental drowning and submersion in a natural body of water | W69 |
| 275 | Accidental drowning and submersion following a fall into a natural  body of water | W70 |
| 276 | Other specified drowning and submersion, unspecified drowning  and submersion | W73-W74 |
| 277 | Accidental inhalation and ingestion resulting in obstruction of  respiratory tract, foreign body entering other orifices | W75-W84 |
| 278 | Accidents caused by fire, smoke, flame | X00-X09 |
| 279 | Accidental poisoning by and exposure to alcohol | X45 |
| 280 | Accidental poisoning by and exposure to drugs, medications, and  biological substances, except narcotics and psychodysleptics | X40,X41,X43,X44 |
| 281 | Accidental poisoning by and exposure to narcotics and  psychodysleptics [hallucinogens], not elsewhere classified | X42 |
| 282 | Other accidental poisonings | X46-X49 |
| 283 | Intentional self-poisoning by and exposure to alcohol | X65 |
| 284 | Other intentional self-harm | X60-X64, X66-X84 |

| 285 | Homicide and injury purposely inflicted by other persons, including legal execution | X85-Y09 |
| --- | --- | --- |
| 286 | Poisoning by and exposure to medication with unknown intentions | Y10-Y11, Y13-Y14 |
| 287 | Poisoning by and exposure to narcotics and psychodysleptics (hallucinogens), not elsewhere classified, undetermined intent | Y12 |
| 288 | Poisoning by and exposure to alcohol, undetermined intent | Y15 |
| 289 | Other poisonings with unknown intentions | Y16-Y19 |
| 290 | Hanging, strangulation, and suffocation, undetermined intent | Y20 |
| 291 | Drowning and submersion, undetermined intent | Y21 |
| 292 | Handgun, rifle, shotgun, and larger firearm discharge,  other and unspecified firearm discharge, undetermined intent | Y22-Y24 |
| 293 | Exposure to smoke, fire, and flames, undetermined intent | Y26 |
| 294 | Contact with a sharp or blunt object, undetermined intent | Y28-Y29 |
| 295 | Falling, jumping, or being pushed from a high place, undetermined intent | Y30 |
| 296 | Motor vehicle collisions, undetermined intent | Y32 |
| 297 | Other specified and unspecified events, undetermined intent | Y33-Y34 |
| 298 | Other damage with uncertain intent | Y25, Y27, Y31 |
| 299 | Operations of war | Y36 |
| 300 | Adverse events experienced by patients during medical care | Y40-Y84 |
| 301 | Accident caused by firearm missile | W32-W34 |
| 302 | Exposure to excessive natural cold | X31 |
| 303 | Exposure to or sequelae of other specified or unspecified factors | X58,X59,Y872,Y899 |
| 304 | Terrorism | Y38, Y892 |
| 305 | Other and unspecified external causes, late effects of external causes | W20-W31,W35-W64,X10- X30,X32-X39,X50-X57,Y35,Y85-  Y871,Y88-Y891,W85-W99 |
| 306 | Severe acute respiratory syndrome | U04 |

*The point separating the fourth symbol is omitted throughout.

# Appendix 3 – Russian oblasts included in the analysis

**Table A4. Official oblast codes and their codes in the mortality database^#^**

| **Code used in the Russian mortality database** | **Name of the region** | **Official region code** |
| --- | --- | --- |
| 3 | Belgorod oblast | 1114 |
| 4 | Bryansk oblast | 1115 |
| 5 | Vladimir oblast | 1117 |
| 6 | Voronezh oblast | 1120 |
| 7 | Ivanovo oblast | 1124 |
| 8 | Kaluga oblast | 1129 |
| 9 | Kostroma oblast | 1134 |
| 10 | Kursk oblast | 1138 |
| 11 | Lipetzk oblast | 1142 |
| 12 | Moscow oblast | 1146 |
| 13 | Oryol oblast | 1154 |
| 14 | Ryazan oblast | 1161 |
| 15 | Smolensk oblast | 1166 |
| 16 | Tambov oblast | 1168 |
| 17 | Tver oblast | 1128 |
| 18 | Tula oblast | 1170 |
| 19 | Yaroslavl oblast | 1178 |
| 20 | Moscow | 1145 |
| 22 | Republic of Karelia | 1186 |
| 23 | Republic of Komi | 1187 |
| 24 | Arkhangelsk oblast | 1111 |
| 25 | Nenets autonomous district | 111 |
| 26 | Vologda oblast | 1119 |
| 27 | Kaliningrad oblast | 1127 |
| 28 | Leningrad oblast | 1141 |
| 29 | Murmansk oblast | 1147 |
| 30 | Novgorod | 1149 |
| 31 | Pskov oblast | 1158 |
| 32 | Sankt-Petersburg | 1140 |
| 34 | Republic of Adygeya | 1179 |
| 35 | Republic of Kalmykia | 1185 |
| 36 | Krasnodar kray | 1103 |
| 37 | Astrakhan oblast | 1112 |
| 38 | Volgograd oblast | 1118 |
| 39 | Rostov oblast | 1160 |
| 41 | Republic of Dagestan | 1182 |
| 42 | Republic of Ingushetia | 1126 |
| 43 | Kabardino-Balkar Republic | 1183 |
| 44 | Karachey-Cherkassian Republic | 1191 |
| 45 | Republic of North Ossetia - Alania | 1190 |
| 46 | Chechen Republic | 1196 |
| 47 | Stavropol kray | 1107 |
| 49 | Republic of Bashkortostan | 1180 |
| 50 | Republic of Mariy El | 1188 |
| 51 | Republic of Mordovia | 1189 |
| 52 | Republic of Tatarstan | 1192 |
| 53 | Udmurt Republic | 1194 |
| 54 | Chuvash Republic | 1197 |
| 55 | Perm kray | 1157 |
| 56 | Kirov oblast | 1133 |
| 57 | Nizhny Novgorod oblast | 1122 |
| 58 | Orenburg oblast | 1153 |
| 59 | Penza oblast | 1156 |
| 60 | Samara oblast | 1136 |
| 61 | Saratov oblast | 1163 |
| 62 | Ulyanovsk oblast | 1173 |
| 64 | Kurgan oblast | 1137 |
| 65 | Sverdlovsk oblast | 1165 |
| 66 | Tyumen oblast | 1171 |
| 67 | Khanty-Mansi autonomous area | 171 |
| 68 | Yamalo-Nenets autonomous area | 271 |
| 69 | Chelyabinsk oblast | 1175 |
| 71 | Republic of Altai | 1184 |
| 72 | Republic of Buryatia | 1181 |
| 73 | Republic of Tuva | 1193 |
| 74 | Republic of Khakasia | 1195 |
| 75 | Altai kray | 1101 |
| 76 | Zabaikalsk kray | 1176 |
| 77 | Krasnoyarsk kray | 1104 |
| 78 | Irkutsk oblast | 1125 |
| 79 | Kemerovo oblast | 1132 |
| 80 | Novosibirsk oblast | 1150 |
| 81 | Omsk oblast | 1152 |
| 82 | Tomsk oblast | 1169 |
| 84 | Republic of Sakha (Yakutia) | 1198 |
| 85 | Kamchatka kray | 1130 |
| 86 | Primorsky kray | 1105 |
| 87 | Khabarovsk kray | 1108 |
| 88 | Amur oblast | 1110 |
| 89 | Magadan oblast | 1144 |
| 90 | Sakhalin oblast | 1164 |
| 91 | Jewish autonomous oblast | 1199 |
| 92 | Сhukotka autonomous area | 1177 |

*^#^* Oblasts included in the consistent analysis of mortality patterns and changes from 2000 to 2018
*Sources:* Russian Fertility and Mortality database (RusFMD) and authors; calculations.

# Appendix 4- Adjustments made for data processing

**Preventable mortality**

Up to 2010

- Codes A49.2, J65, J82, and J92 were included within large groups of diseases and thus were not evaluated in the analysis.
- Code C45 was included in a broad category of diseases (C45–C49) and thus was not included in the analysis.
- Aortic aneurysms were also included as part of a large group of diseases (I71-I79); however, we chose to include this diagnosis in the analysis.

From 2011 onwards

- A49.2, J65, and C45 were included together with their respective larger groups of diseases and thus were excluded from the analysis.
- Aortic aneurysms were included as part of a large group of diseases (I71-I79) but were considered in the analysis.

**Treatable deaths**

Up to 2010

- Code J65 was included within a large group of diseases (codes J60–J70) and was not evaluated as part of the death count.
- Code L03 was included together with all causes of death (codes L00–L98 and thus was not evaluated in the analysis.
- Codes A48.1 and A49.1 were included in the category “Other bacterial infections excluding foodborne illnesses/toxins” and thus were not considered in the analysis.
- Code C62 (testicular cancer) was included in the category “Other diseases of male genital organs” and was not evaluated in the analysis/
- Code C73 was included together with a large group of other diseases in the category “Malignant neoplasms of other and independent (primary) multiple sites” and was not evaluated in the analysis.
- Lymphoid leukemia was included together with all leukemia-related diseases and was not evaluated in the analysis.
- Codes D10–D36 are diseases that are presented as part of a larger group of disorders that includes other neoplasms; these have all been included in the analysis.
- Thyroid disorders (E00–E07) are included in a category with two other diseases (“Diseases of other endocrines glands”); these codes have been included in the analysis.
- Aortic aneurysms are included as part of a large group of diseases (I71–I79) and have been included in the analysis.
- I73.9 is included in the category listed just above.
- Codes J90, J93, and J94 were included in a larger group of diseases (J30–39, J90–99) and were not considered in the analysis.
- Codes K82–K83 were not considered in the analysis because they were included in a larger group of diseases categorised as “Other diseases of the digestive system”.
- Inflammatory diseases of the genitourinary system were not considered because they were included in a larger group of diseases categorised as “Other diseases of genital organs”.

From 2011 onwards

- Code J65 was included in a larger group of diseases and thus was not considered in the analysis.
- Code L03 was included together with all causes of death (L00–L08) and thus was not considered in the analysis.
- Codes A48.1 and A49.1 were included together with a larger group of diseases categorised as “Other bacterial infections excluding foodborne illnesses/toxins” and were not considered in the analysis.
- Code C62 (testicular cancer) was included in a category together with two other diseases referred to as “Diseases of male genital organs”. Diseases assigned to this code were considered in the analysis.
- The diagnosis of lymphoid leukemia was included together with all leukemia-related diseases and thus was not considered in the analysis.
- Codes D10–D36 were part of a larger group of diseases that included other neoplasms, however, we did consider them in the analysis.
- Thyroid disorders (E00–E07) were included in a category with two other diseases (“Diseases of other endocrines glands”) however we considered them in the analysis.
- Codes J90, J93, and J94 were included in a larger group of diseases (J30–39, J90–99) and thus were not considered in the analysis.
- Codes K82–K83 were included as part of the larger category “Other diseases of the digestive system” and thus were not considered in the analysis.
- Inflammatory diseases of the genitourinary system were not considered in the analysis as they were included as part of a larger group categorised as “Other diseases of genital organs”.

# Appendix 5 - Additional variables used in the modelling exercise

*Healthcare infrastructure*

In order to account for the availability of health care, we included three variables, i.e., the availability of physicians, nurses, and hospital beds, respectively, per 10,000 inhabitants. Data for these variables were obtained directly from the Rosstat regional dataset.

*Other variables that capture socio-economic development on a regional level*

We also included variables that reflected the level of socioeconomic development on a regional basis. These variables included (i) gross regional product (GRP) per capita; (ii) growth of the GRP per capita; (iii) poverty rate; (iv) density of the population per square kilometre; (v) proportion of the population residing in urban centres; and (vi) the female to male ratio. Data for these variables were obtained from Rosstat regional tables.

# Appendix 6 – Additional tables and figures

**Figure A1. Treatable mortality rates for males and females in the Russian Federation.** Data are presented as treatable deaths per 100,000 person-years.

*Sources:* Russian Fertility and Mortality database (RusFMD), OECD/Eurostat, and the authors’ calculations. The cut-off point is 75 years, as per the international literature.

**Figure A2. Preventable mortality rates and mortality rates due to causes not considered preventable in the Russian Federation.** Data are presented per 100,000 person-years.

*Sources:* Russian Fertility and Mortality database (RusFMD), OECD/Eurostat, and the authors’ calculations. The cut-off point is 75 years as per the international literature.

**Figure A3. Treatable mortality rates and mortality rates due to causes not considered treatable, for males and females in the Russian Federation.** Data are presented as treatable deaths per 100,000 person-years.

*Sources:* Russian Fertility and Mortality database (RusFMD), OECD/Eurostat, and the authors’ calculations. The cut-off point is 75 years as per the international literature.

**Table A5. Definitions of variables used in the analysis**

|  | **Type of variable** | **Definition** |
| --- | --- | --- |
| Sales of vodka per inhabitant | Continuous | total sales of vodka (dcl) per oblast; this value was normalised using the population of each oblast |
| Smoking (% of the adult population) | Continuous | Share of the adult population that has practiced past, occasional, and daily tobacco use |
| Population density (/km^2^) | Continuous | Total number of inhabitants per one kilometre squared. |
| Urban population (% of total) | Continuous | Share of the overall population per oblast that is urban. |
| Gross regional product (GRP) per capita | Continuous | Regional value-added expressed per inhabitant and also expressed in real terms. |
| Poverty (% of the population) | Continuous | Share of the overall population per oblast that lives below the national poverty line |
| Female to male ratio | Continuous | Number of females per 1000 males. |
| Physicians per 10,000 inhabitants | Continuous | Total number of physicians per 10,000 oblast inhabitants |
| Hospital beds per 10,000 inhabitants | Continuous | Total number of hospital beds per 10,000 oblast inhabitants |
| Nurses per 10,000 inhabitants | Continuous | Total number of nurses per 10,000 oblast inhabitants |
| Health expenditures (% of GRP) | Continuous | Overall health expenditure per oblast expressed as a share of the overall value added. |

**Table A6. Treatable and preventable mortality over a more limited age range (ages 0 –70 and 0 –65 years).** Shown are the number of cases (per 100,000 person-years) and the percent change from data based on the original upper age limit of 75 years.

| **Age limit of 75 years** | | | **Age limit of 70 years** | | **Age limit of 65 years** | |  | **Difference: 75 to 70** | | **Difference: 75 to 65** | |
| --- | --- | --- | --- | --- | --- | --- | --- | --- | --- | --- | --- |
| **Year** | **Treatable**  **(# per 100,000)** | **Preventable**  **(# per 100,000)** | **Treatable**  **(# per 100,000)** | **Preventable**  **(# per 100,000)** | **Treatable**  **(# per 100,000)** | **Preventable**  **(# per 100,000)** | | **Treatable**  **(%)** | **Preventable**  **(%)** | **Treatable**  **(%)** | **Preventable**  **(%)** |
| **2000** | 337.2 | 548.6 | 275.7 | 478.9 | 220.9 | 412.8 |  | 22.3 | 14.5 | 52.6 | 32.9 |
| **2001** | 338.2 | 556.7 | 276.5 | 487.0 | 222.1 | 421.5 |  | 22.3 | 14.3 | 52.3 | 32.1 |
| **2002** | 346.1 | 570.2 | 282.3 | 498.3 | 227.3 | 432.2 |  | 22.6 | 14.4 | 52.3 | 31.9 |
| **2003** | 347.4 | 569.4 | 283.6 | 498.2 | 229.8 | 434.5 |  | 22.5 | 14.3 | 51.2 | 31.0 |
| **2004** | 331.4 | 548.2 | 270.8 | 480.7 | 219.1 | 420.0 |  | 22.4 | 14.0 | 51.2 | 30.5 |
| **2005** | 329.3 | 539.3 | 269.5 | 473.2 | 218.9 | 413.8 |  | 22.2 | 14.0 | 50.5 | 30.3 |
| **2006** | 299.4 | 517.3 | 242.6 | 455.2 | 195.1 | 399.7 |  | 23.4 | 13.6 | 53.5 | 29.4 |
| **2007** | 279.6 | 480.6 | 226.8 | 422.8 | 180.7 | 368.7 |  | 23.3 | 13.7 | 54.7 | 30.3 |
| **2008** | 275.3 | 467.5 | 224.4 | 412.3 | 178.1 | 358.4 |  | 22.7 | 13.4 | 54.6 | 30.4 |
| **2009** | 263.0 | 438.3 | 213.1 | 383.8 | 169.0 | 332.2 |  | 23.4 | 14.2 | 55.6 | 31.9 |
| **2010** | 257.9 | 430.0 | 208.1 | 376.1 | 164.2 | 325.3 |  | 23.9 | 14.3 | 57.0 | 32.2 |
| **2011** | 242.6 | 389.1 | 196.4 | 339.6 | 156.9 | 293.9 |  | 23.5 | 14.6 | 54.6 | 32.4 |
| **2012** | 233.6 | 371.6 | 187.4 | 322.4 | 150.3 | 280.1 |  | 24.6 | 15.3 | 55.5 | 32.7 |
| **2013** | 223.8 | 357.4 | 179.0 | 310.0 | 143.4 | 269.7 |  | 25.0 | 15.3 | 56.1 | 32.5 |
| **2014** | 216.4 | 356.2 | 174.0 | 311.0 | 138.5 | 270.4 |  | 24.4 | 14.5 | 56.3 | 31.7 |
| **2015** | 207.7 | 342.9 | 166.5 | 299.3 | 131.1 | 258.7 |  | 24.7 | 14.6 | 58.4 | 32.6 |
| **2016** | 199.2 | 330.7 | 160.3 | 289.8 | 124.9 | 248.7 |  | 24.2 | 14.1 | 59.5 | 33.0 |
| **2017** | 184.9 | 306.0 | 149.0 | 268.0 | 115.2 | 229.1 |  | 24.1 | 14.2 | 60.4 | 33.6 |
| **2018** | 183.4 | 301.3 | 147.9 | 264.2 | 114.0 | 225.3 |  | 23.9 | 14.0 | 60.8 | 33.7 |

**Table A7. Treatable mortality and contributing causes amongst males and females in the Russian Federation.**

| **Males** | | | | | | |
| --- | --- | --- | --- | --- | --- | --- |
| **Treatable mortality; # per 100,000 person-years** | | |  | **Percent of overall treatable mortality** | | |
|  | **2000** | **2018** |  |  | **2000** | **2018** |
| Tuberculosis (TB) | 19.5 | 4.6 |  | Tuberculosis (TB) | 4.1 | 1.8 |
| Other infectious diseases | 3.6 | 1.8 |  | Other infectious diseases | 0.8 | 0.7 |
| Cancers | 25.0 | 22.7 |  | Cancers | 5.3 | 8.8 |
| Diabetes | 2.5 | 6.3 |  | Diabetes | 0.5 | 2.4 |
| Ischaemic Heart Disease (IHD) | 182.6 | 103.3 |  | Ischaemic Heart Disease (IHD) | 38.4 | 40.1 |
| Hypertensive disorders | 7.8 | 3.1 |  | Hypertensive disorders | 1.6 | 1.2 |
| Cerebrovascular diseases | 99.5 | 45.3 |  | Cerebrovascular diseases | 20.9 | 17.6 |
| Respiratory | 56.1 | 23.7 |  | Respiratory | 11.8 | 9.2 |
| Nephritis | 3.4 | 0.9 |  | Nephritis | 0.7 | 0.3 |
| **Female** | | | | | | |
| **Treatable mortality; # per 100,000 person-years** | | |  | **Percent of overall treatable mortality** | | |
|  | **2000** | **2018** |  |  | **2000** | **2018** |
| Tuberculosis (TB) | 2.3 | 0.9 |  | Tuberculosis (TB) | 1.0 | 0.7 |
| Other infectious diseases | 1.9 | 1.1 |  | Other infectious diseases | 0.8 | 0.9 |
| Cervical cancer | 3.0 | 3.2 |  | Cervical cancer | 1.3 | 2.4 |
| Other cancers | 43.2 | 34.9 |  | Other cancers | 18.4 | 26.9 |
| Diabetes | 3.6 | 6.1 |  | Diabetes | 1.5 | 4.7 |
| Ischaemic Heart Disease (IHD) | 60.9 | 31.4 |  | Ischaemic Heart Disease (IHD) | 25.9 | 24.2 |
| Hypertensive disorders | 5.2 | 1.6 |  | Hypertensive disorders | 2.2 | 1.2 |
| Cerebrovascular diseases | 57.5 | 19.7 |  | Cerebrovascular diseases | 24.4 | 15.2 |
| Respiratory | 15.3 | 7.1 |  | Respiratory | 6.5 | 5.4 |
| Nephritis | 1.9 | 0.5 |  | Nephritis | 0.8 | 0.4 |
| Pregnancy-related mortality | 0.7 | 0.2 |  | Pregnancy-related mortality | 0.3 | 0.1 |

*Sources:* Russian Fertility and Mortality database (RusFMD), OECD/Eurostat, and authors’ calculations.

**Figure A4. Preventable mortality at the subnational level.** Shown are data from the years 2018 for males (A) and females (B) in the Russian Federation. Data are presented as preventable deaths per 100,000 person-years.

**Panel A**


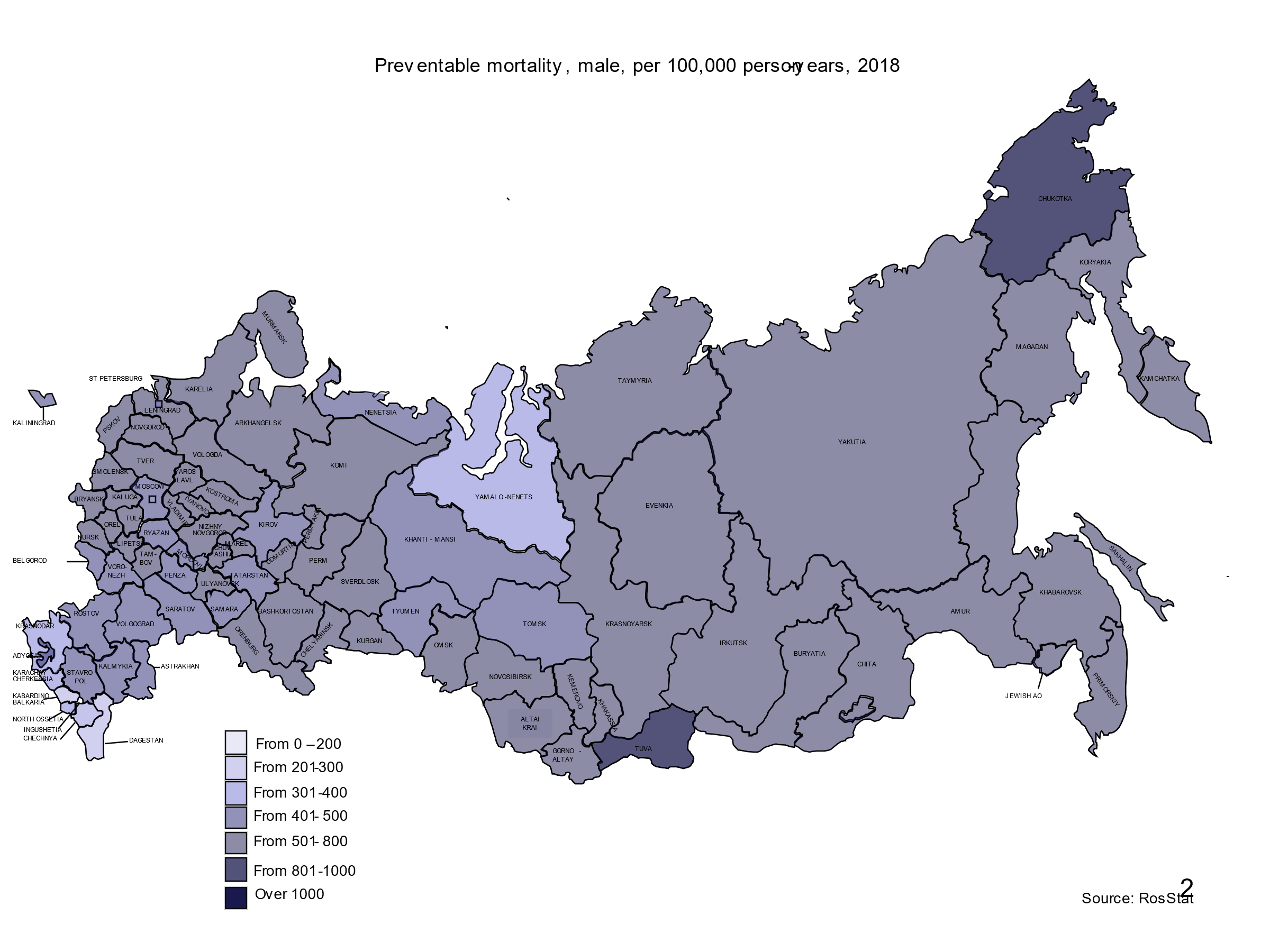


**Panel B**


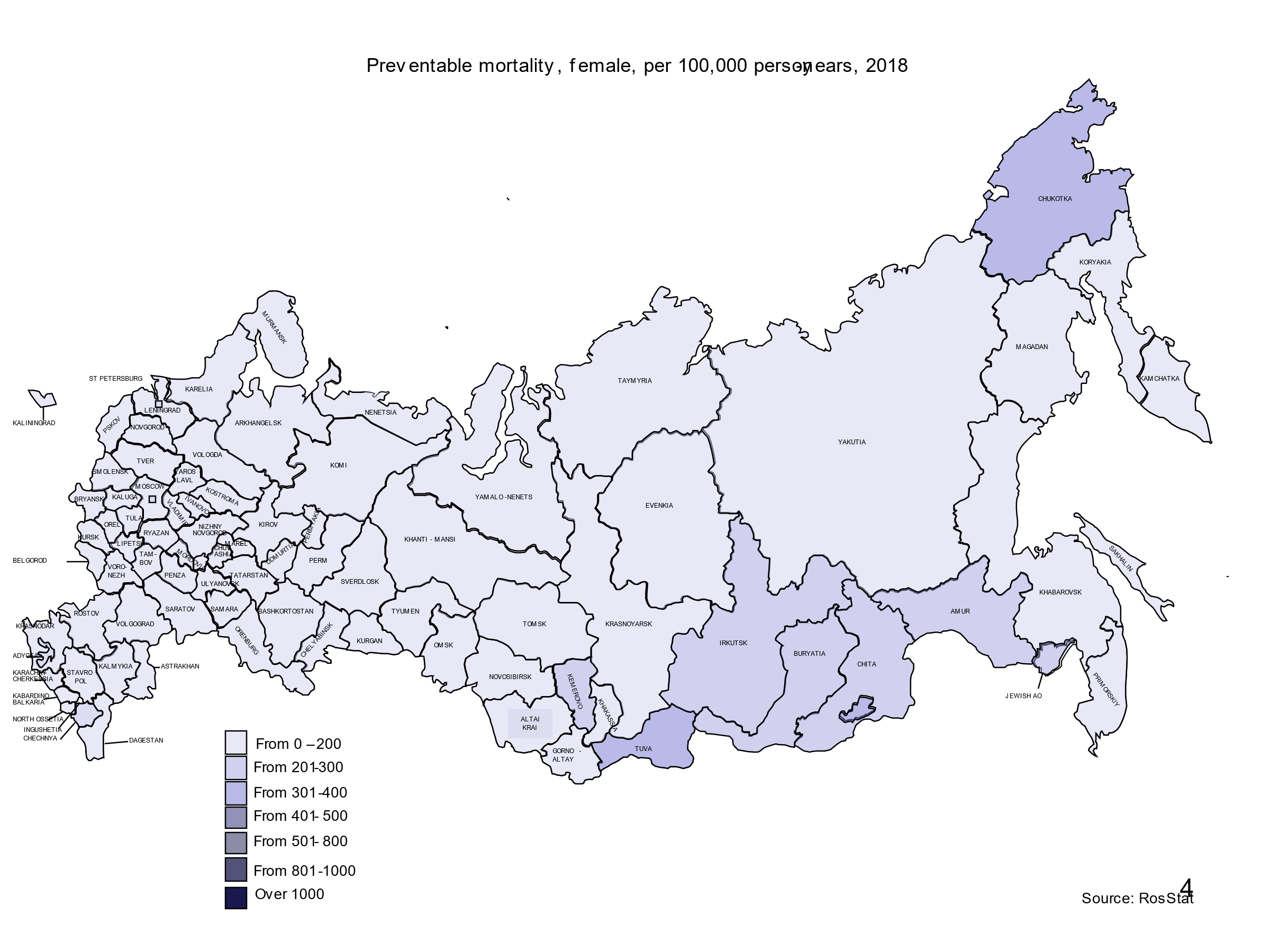


**Figure A5. Reduction in overall preventable mortality at the subnational level.** Data are presented as percentage change.


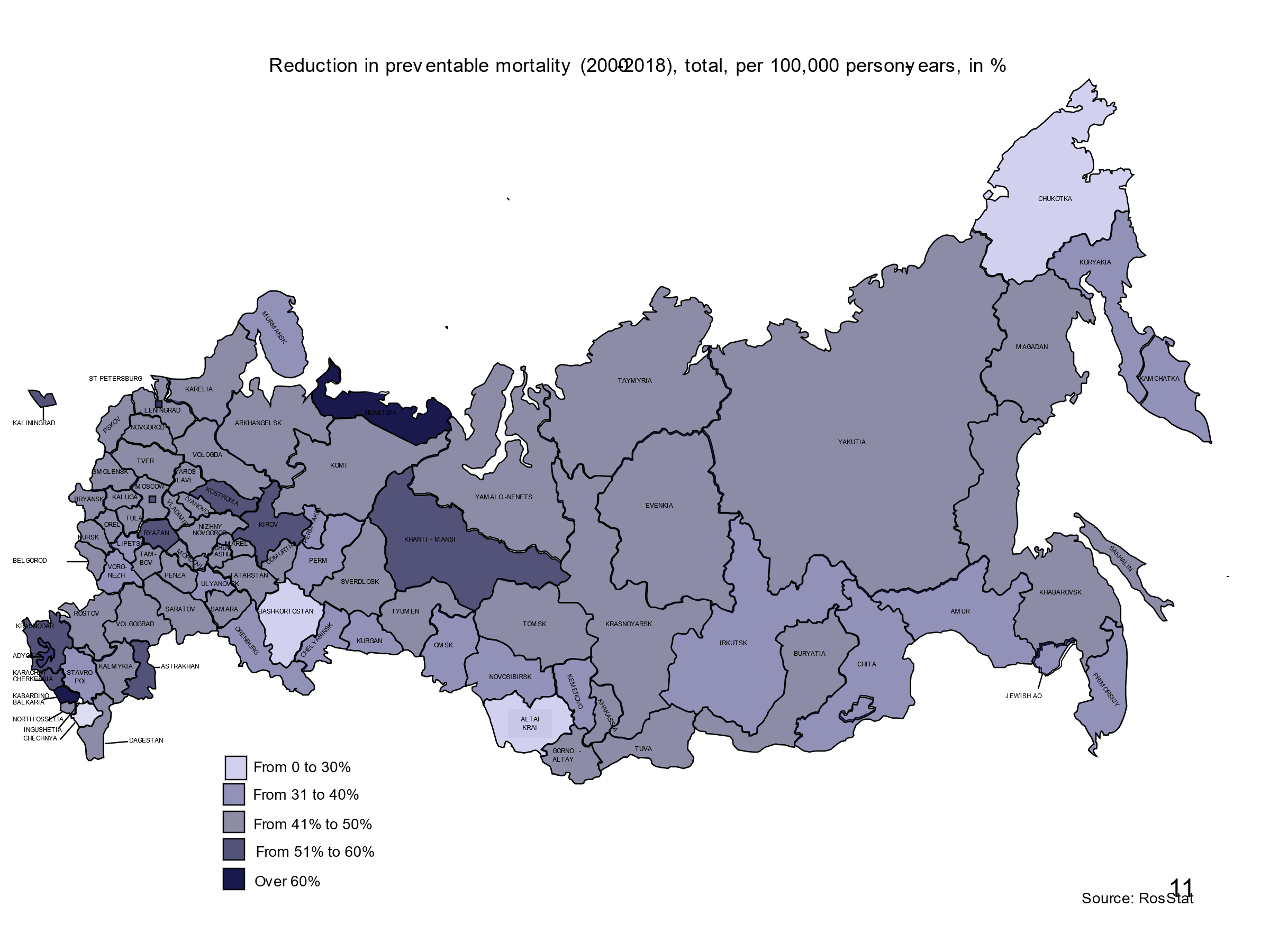


**Figure A6. Overall preventable mortality at the subnational level.** Shown are data from the year 2018. Data are presented as preventable deaths per 100,000 person-years.


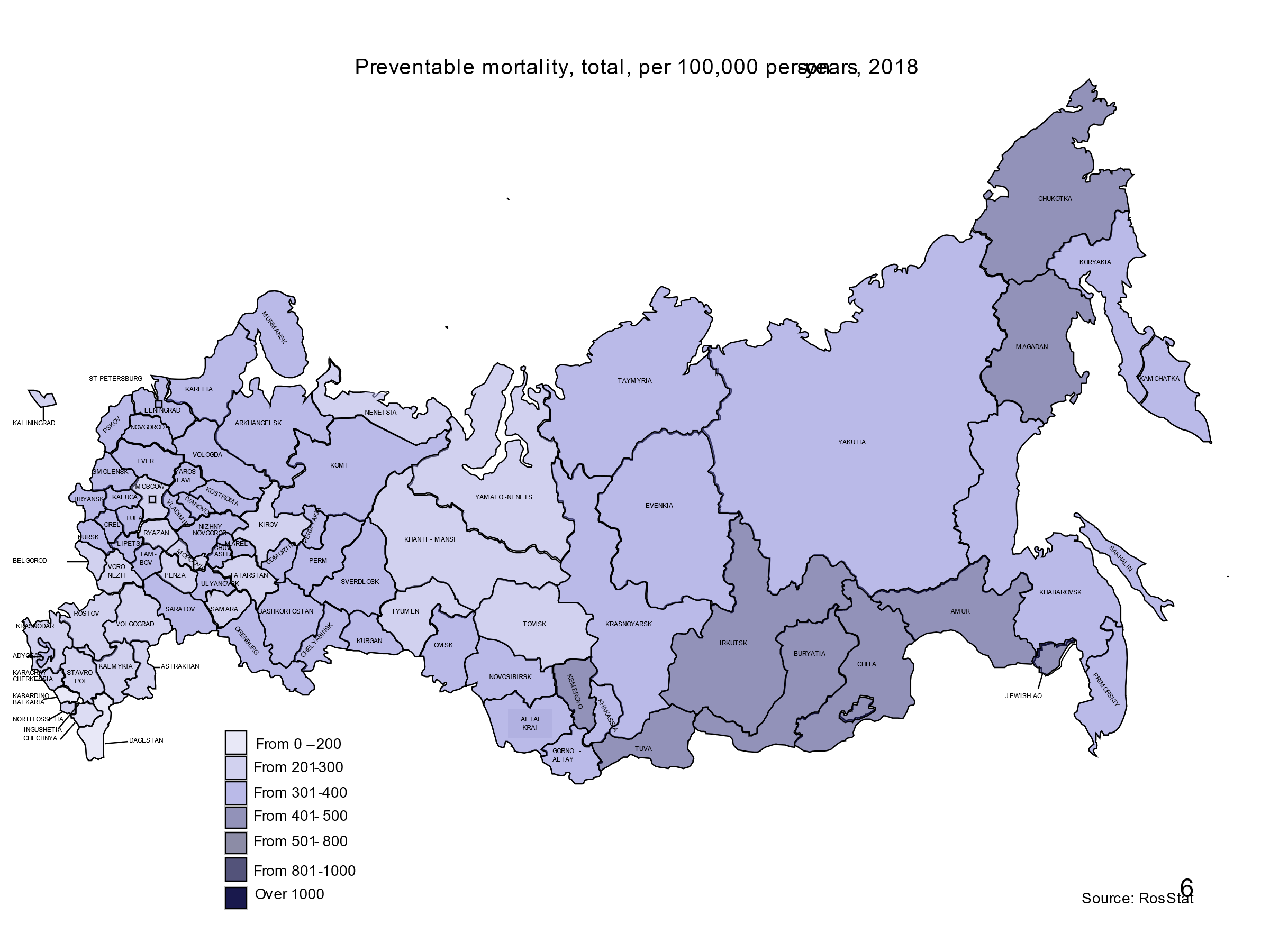


**Figure A7. Reduction in treatable mortality at the subnational level.** Shown are data for males. Data are presented as percentage change.


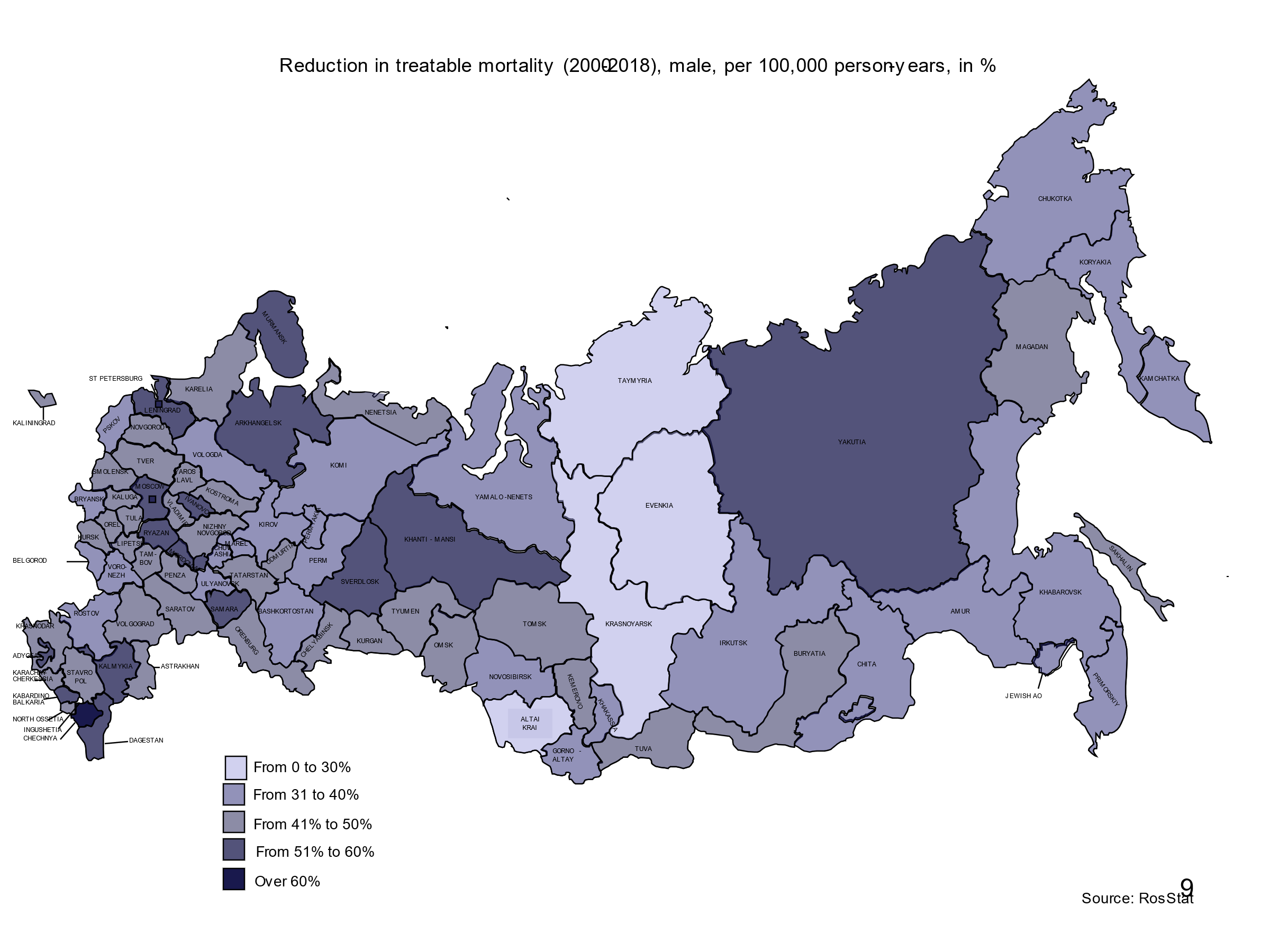


**Figure A8. Reduction in treatable mortality at the subnational level.** Shown are data for females. Data are presented as percentage change.


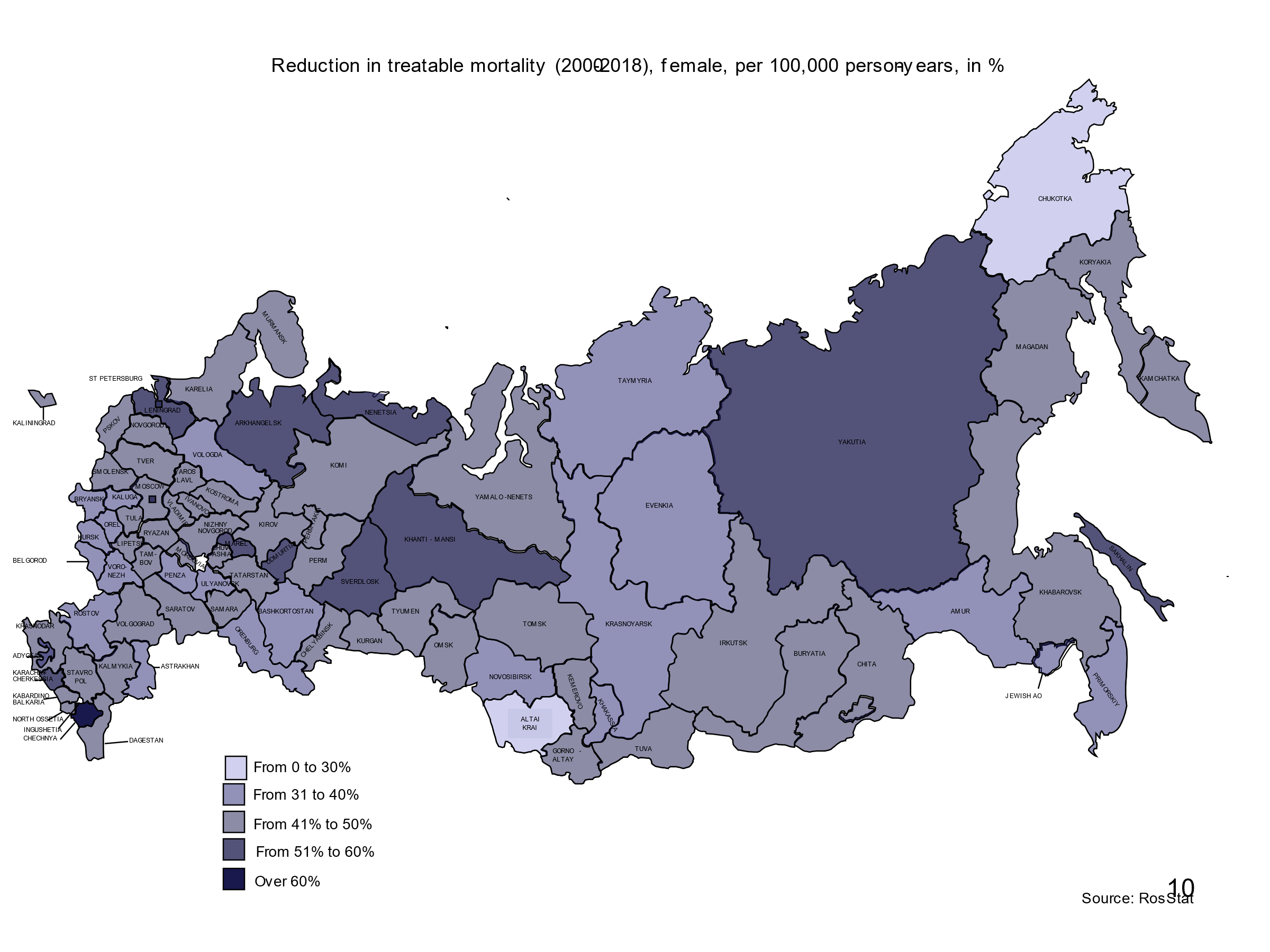


**Figure A9. Change in treatable mortality at the subnational level.** Shown are data for both genders. Data are presented as percentage change.


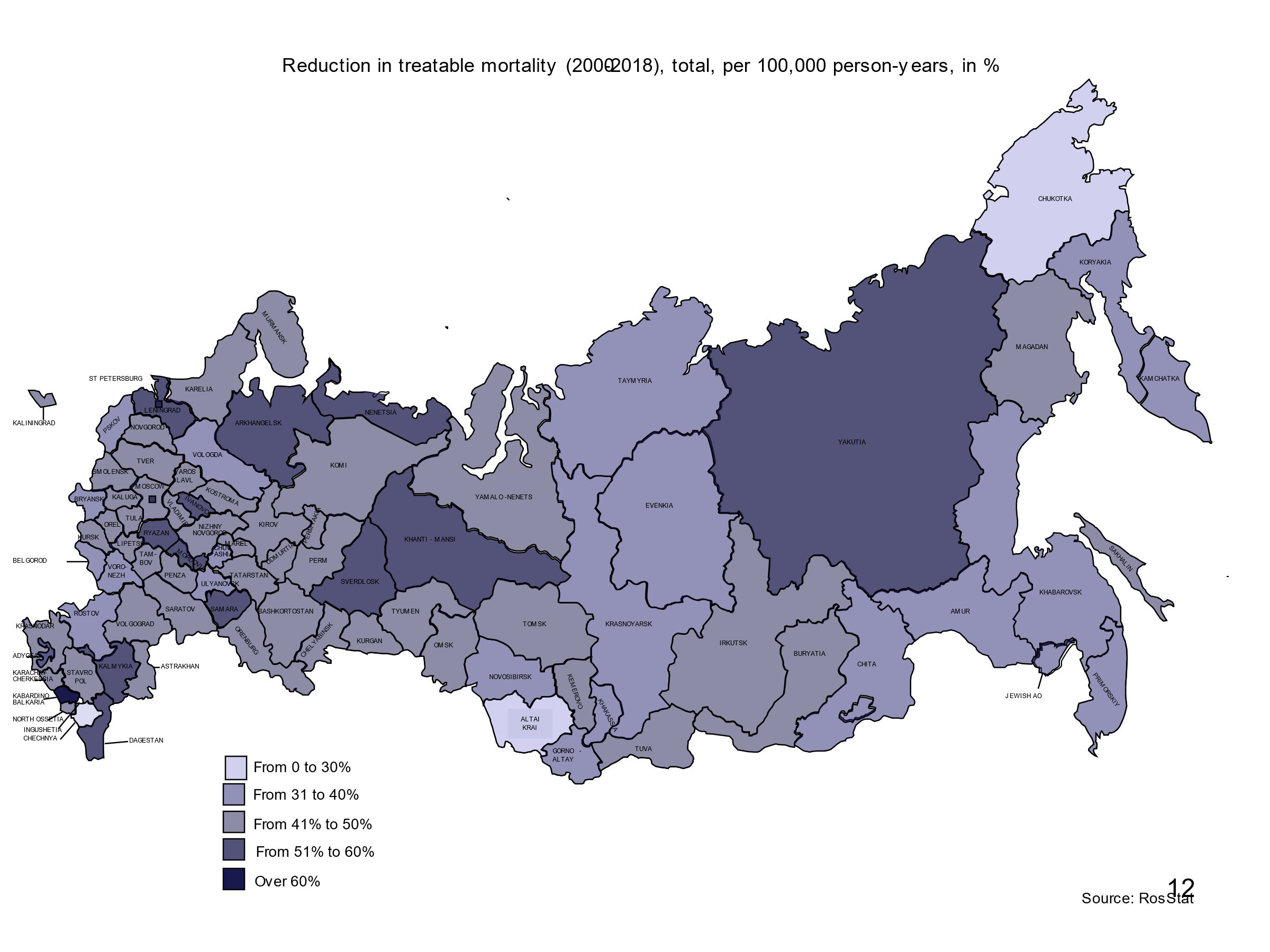


**Figure A10. Treatable mortality at the subnational level.** Shown are data from 2018 for males (Panel A) and females (Panel B). Data are presented as treatable deaths per 100,000 male or female (respectively) person-years in the Russian Federation.

**Panel A**

**
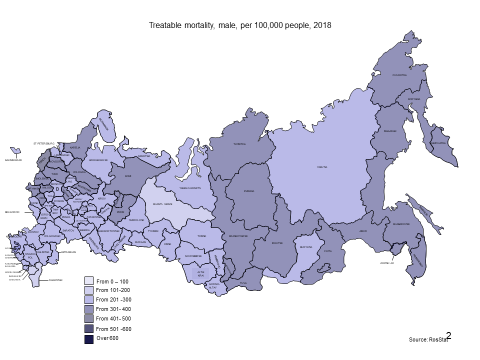
**

**Panel B**


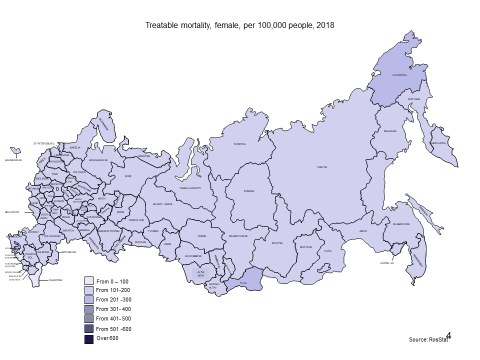


**Figure A11. Overall treatable mortality at the subnational level.** Shown are data for 2018. Data are presented as treatable deaths per 100,000 person-years in the Russian Federation.


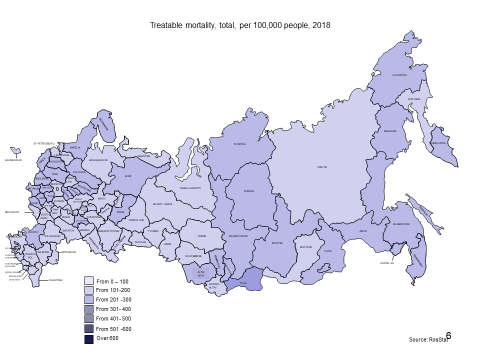


**Figure A12. Correlation between preventable mortality rates and mortality rates to causes not considered preventable in the Russian Federation.** Data are presented for males (A) and females (B) per 100,000 person-years.

**A.**

**B.**

*Sources:* Russian Fertility and Mortality database (RusFMD), OECD/Eurostat, and the authors’ calculations

**Figure A13. Correlation between treatable mortality rates and mortality rates to causes not considered treatable in the Russian Federation.** Data are presented for males (A) and females (B) per 100,000 person-years.

**A.**

**B.**

*Sources:* Russian Fertility and Mortality database (RusFMD), OECD/Eurostat, and the authors’ calculations

**Table A8. Summary of variables used in the analysis**

|  | Number of Observations | Years included in the analysis | Mean | Standard Deviation | Minimum | Maximum |
| --- | --- | --- | --- | --- | --- | --- |
| Overall preventable mortality (log_10_) | 249 | 2014, 2016, 2018 | 5.8 | 0.3 | 4.4 | 6.5 |
| Sales of vodka (as a % of total alcohol sales) | 248 | 2014, 2016, 2018 | 9.9 | 5.6 | 0.1 | 57.4 |
| Smokers (% of the total population) | 249 | 2014, 2016, 2018 | 37.2 | 8.6 | 9.3 | 76.0 |
| Population density (/km^2^) | 249 | 2014, 2016, 2018 | 131.4 | 665.2 | 0.1 | 5023.9 |
| Urban population (% of total) | 249 | 2014, 2016, 2018 | 70.4 | 13.1 | 29.2 | 100.0 |
| Gross regional product (GRP) per capita (log_10_) | 249 | 2014, 2016, 2018 | 11.2 | 0.7 | 9.8 | 14.0 |
| Poverty (%) | 249 | 2014, 2016, 2018 | 14.4 | 5.1 | 5.8 | 37.8 |
| Female to male ratio | 249 | 2014, 2016, 2018 | 1144.0 | 53.5 | 961.0 | 1233.0 |
| Physicians per 10,000 inhabitants | 249 | 2014, 2016, 2018 | 47.2 | 9.0 | 26.5 | 81.5 |
| Hospital beds per 10,000 inhabitants | 249 | 2014, 2016, 2018 | 87.6 | 15.0 | 44.4 | 152.0 |
| Nurses per 10,000 inhabitants | 249 | 2014, 2016, 2018 | 111.1 | 16.8 | 70.9 | 167.1 |
| Health expenditures (% of GRP) | 249 | 2014, 2016, 2018 | 2.2 | 1.4 | 0.1 | 7.9 |

*Sources:* Russian Fertility and Mortality database (RusFMD), OECD/Eurostat, Rosstat, and the authors’ calculations.

**Table A9. Results of fixed effects regression analysis using log_10_ of preventable mortality as a dependent variable**

| **Variable** | **Model 1** | **Model 2** | **Model 3** |
| --- | --- | --- | --- |
|  |  |  |  |
| Sales of vodka (as a % of total alcohol sales) | -0.008 (0.001) |  | -0.009 (0.001) |
| Current smokers, % | 0.003* (0.001) |  | 0.003 (0.001) |
| Population density, per km^2^ | 0.0005** (0.0002) | 0.004*** (0.001) | 0.0005*** (0.0001) |
| Urban population, % | -0.006 (0.003) | -0.005** (0.002) | -0.003 (0.003) |
| Gross regional product (GRP) per capita, log_10_ | 0.022 (0.057) | 0.022 (0.048) | 0.043 (0.048) |
| Poverty, % | -0.003 (0.003) | -0.003 (0.003) | -0.003 (0.003) |
| Ratio of female to male inhabitants | 0.0008 (0.0007) | 0.001** (0.0007) | 0.001** (0.0006) |
| Physicians per 10,000 inhabitants |  | -0.00003 (0.002) | -0.00002 (0.002) |
| Hospital beds per 10,000 inhabitants |  | 0.001 (0.001) | 0.001 (0.001) |
| Nurses per 10,000 inhabitants |  | -0.003** (0.001) | -0.003* (0.001) |
| Health expenditures, % of GRP |  | 0.001 (0.0007) | 0.001 (0.005) |
| Intercept | 5.02*** (1.31) | 4.35*** (1.06) | 4.04*** (1.11) |
|  |  |  |  |
| R squared | 0.078 | 0.104 | 0.070 |
| Number of observations | 248 | 249 | 248 |
| Year effects | yes | yes | yes |
| Groups | 83 | 83 | 83 |
| ****p* <0.01; ***p* <0.05; **p*< 0.10. The values reported here are parameter estimates from three independent regression analyses. All models were estimated with robust standard errors that are reported in parentheses. | | | |
| The models also control for the following variables: GRP per capita, population density (per km^2^), percentage of the population living in an urban setting, poverty rates, and the ratio of female to male inhabitants. | | | |

*Sources:* Russian Fertility and Mortality database (RusFMD), OECD/Eurostat, Rosstat, and the authors’ calculations.

**Table A10. Results of fixed effects regression analysis using log_10_ of treatable mortality as a dependent variable**

| **Variable** | **Model 2** |
| --- | --- |
|  |  |
| Population density, per km^2^ | 0.003** (0.001) |
| Urban population, % | 0.004** (0.002) |
| Gross regional product (GRP) per capita, log_10_ | -0.047 (0.048) |
| Poverty, % | 0.001 (0.003) |
| Ratio of female to male inhabitants | 0.0001 (0.0007) |
| Physicians per 10,000 inhabitants | -0.001 (0.002) |
| Hospital beds per 10,000 inhabitants | 0.0006 (0.001) |
| Nurses per 10,000 inhabitants | -0.002 (0.001) |
| Health expenditures, % of GRP | 0.008 (0.004) |
| Intercept | 5.59*** (1.05) |
|  |  |
| R squared | 0.02 |
| Observations | 253 |
| Year effects | yes |
| Groups | 85 |
| ****p* <0.01; ***p* <0.05; **p*< 0.10. The values reported here are parameter estimates from independent regression analyses. All models were estimated with robust standard errors that are reported in parentheses. | |
| The models also control for the following variables: GRP per capita, population density (per km^2^), percentage of the population living in an urban setting, poverty rates, and the ratio of female to male inhabitants. | |

*Source:* Russian Fertility and Mortality database (RusFMD), OECD/Eurostat, Rosstat, and the authors’ calculations
